# Exposure to electronic cigarette aerosols triggers alterations in genomic DNA methylation that impacts cancer pathways in mice

**DOI:** 10.1080/08958378.2026.2649516

**Published:** 2026-04-23

**Authors:** Christina Awada, Catherine B. Klein, Terry Gordon, Amna Raja, Kent E. Pinkerton, Morgan Poindexter, Emma Karey, Arul Veerappan, Judith T. Zelikoff

**Affiliations:** a Division of Environmental Medicine, New York University Grossman School of Medicine, New York, NY, USA; b Center for Health and the Environment, University of California, Davis, Davis, CA, USA

**Keywords:** Electronic cigarettes, DNA methylation, cancer epigenetics, inhalation toxicology, lung epigenome

## Abstract

**Objective:**

The aim of this laboratory study was to characterize the effects of sub-chronic electronic cigarette (E-cig) aerosol exposures on genome-wide DNA methylation in mice to begin to elucidate the pathophysiology of E-cig-related pulmonary diseases and cancer.

**Materials and Methods:**

Male C57BL/6 and FVBN mice were randomly assigned to one of two treatment groups (n = 6 per group) and exposed daily to either filtered air or a 50:50 mixture of propylene glycol and vegetable glycerol containing 24 mg/mL nicotine (+Nic). Whole-body inhalation exposures were conducted for 3 h/d, 5 d/week, for a total of 1-month. Sequences for ~285 000 CpG probes were aligned to the mouse genome, and mixed linear models were used to model DNA methylation levels (β values). These evaluations were followed by ingenuity pathway analysis (IPA), Kyoto Encyclopedia of Genes and Genomes (KEGG), and Gene Ontology (GO) analysis.

**Results and Discussion:**

E-cig inhalation exposure induced significant DNA methylation changes in adult male mice, with a notable impact on cancer-related pathways. A total of 2300 genes in C57BL/6 mice and 6732 genes in FVBN mice were hypomethylated, while 1673 and 5529 genes were hypermethylated, respectively. KEGG and GO analyses highlighted key pathways such as Wnt/β-catenin signaling and proteoglycans in cancer, suggesting that E-cig aerosol exposure could disrupt critical genomic regulation and potentially promote carcinogenesis. These in vivo findings underscore the potential cancer-promoting effects of E-cig aerosols through epigenetic modifications.

**Conclusions:**

Findings from this study provide compelling evidence that sub-chronic E-cig exposure induces genomic DNA methylation changes linked to cancer pathways in two strains of adult male mice, highlighting the serious adverse consequences of E-cig use and strain-specific response differences.

## Introduction

Indisputable evidence of the devastating health effects of conventional cigarettes has led to a change in tobacco smoking behavior over the past decade. This shift in smoking behavior has encouraged the use of devices such as electronic cigarettes (E-cigs) and other ENDSs (electronic nicotine delivery systems) products. Electronic cigarettes are battery-powered vaporizers that heat a liquid solution of propylene glycol/vegetable glycerol (PG/VG) usually mixed with nicotine, flavorings, and other additives to deliver an inhaled aerosol (American Lung Association 2024; CDC 2024a). E-cigs have been promoted by the manufacturers as healthier alternatives to traditional cigarettes and as a tool for smoking cessation (CDC 2024a, 2024b). As a result, the prevalence of vaping has reached epidemic proportions, with global usage of E-cigs soaring from 7 million users in 2011 to 82 million in 2021 (Li et al. 2022; Zamora Goicoechea et al. 2024). Concerns regarding the safety and long-term effects of E-cigs on human health persist, particularly concerning their impact on genetic and epigenetic processes (Payne et al. 2016).

Propylene glycol and vegetable glycerin (VG) have historically been considered nontoxic by ingestion (Li et al. 2020). However, evidence demonstrates the formation and release of harmful thermal degradation byproducts when PG/VG solutions are heated. For example, aldehydes such as formaldehyde, acetaldehyde, and acrolein have been detected in E-cig emissions (Ogunwale et al. 2017) and short-term animal studies have revealed modest respiratory effects from inhaling PG and VG aerosols without nicotine or added chemical flavors (Gordon et al. 2022). To date, over 250 chemical compounds, such as nicotine, flavorings, alkaloids, volatile organic compounds (VOCs), pyridine, carbonyl compounds, and metals, have been identified in E-cig aerosols. The heating element within an E-cig is the most significant source of toxic metal exposure, releasing quantifiable metals such as chromium, nickel, selenium, and aluminum (Gordon et al. 2022). In addition, user-controlled alterations and manipulation of power settings in E-cig devices can increase the aerosol concentrations of some of these chemicals. These facts raise concerns regarding the chemical changes that e-liquid ingredient mixtures can undergo during vaping (Wang L et al. 2022). The evidence contributes to the concerns regarding E-cig use and whether it will produce detrimental health effects similar to traditional cigarette smoking exposures. To address this key question, the present study investigated the effects of sub-chronic exposure to E-cig aerosols on the DNA methylome in a rodent model.

Epigenetic studies provide insights into genome interactions with the environment to better understand how lifestyle choices, environmental exposures, and life experiences can affect gene expression over the life course. Numerous studies have shown that exposure to traditional cigarette smoke adversely affects the human epigenome; cigarette smoke-induced alterations in DNA methylation, histone modifications, and non-coding RNAs affect a variety of molecular and cellular processes, including carcinogenesis (Hikisz and Jacenik 2023). Epigenome-wide association studies (EWASs) confirmed that alterations in DNA-associated methylation at gene-specific CpG sites are induced by cigarette smoke (Harlid et al. 2014; Joehanes et al. 2016; Richmond et al. 2021; Mori et al. 2022). Other human studies revealed that expression of DNA methyltransferases (DNMTs), specifically DNMT1, was significantly greater in lung tissue of cigarette smokers compared to its expression in non-cigarette smokers (Wang J et al. 2012; Lee and Pausova 2013; Xiao et al. 2015). Taken together, these studies demonstrated that cigarette smoke induces gene hypermethylation by upregulating DNMT1, leading to the downregulation of certain target genes. Cigarette smoking has also been shown to induce widespread epigenetic alterations, such that DNA methylation profiling can potentially predict smoking behavior (Vidaki et al. 2023). Epigenetics research examines heritable changes in the genome and gene expression patterns that do not involve direct alterations to the DNA sequence. These changes include posttranslational modifications to DNA-bound histone proteins, DNA methylation, chromatin remodeling, and RNA modifications (Zhang et al. 2024) that play crucial roles in development, cellular differentiation, genome stability, and responses to environmental exposures. Gene expression regulation by 5-methyl cytosine (5mC) typically occurs at CpG islands in gene promoters and/or in other sites in gene bodies (Zhang et al. 2024) such that hypermethylation refers to increased DNA methylation, often leading to gene silencing. In contrast, hypomethylation of gene promoters is generally correlated with activation of gene expression or increased genomic instability (Choi and Lee 2013). Since epigenetic modifications are sensitive to, and reversible by, environmental exposures, studying such alterations offer a promising avenue for clinical research and disease treatment.

Research on the epigenetic effects of vaping is limited. Nevertheless, a few animal studies have indicated that E-cig exposure can lead to global DNA methylation changes and cognitive impairments. Specifically, inhalation of E-cig aerosols has led to global hypomethylation of long interspersed nucleotide element 1 (LINE-1) repeats, a marker of overall DNA methylation, which is associated with poor cancer prognosis (Caliri et al. 2020; Kosumi et al. 2020). Moreover, some individual E-cig constituents are associated with inducing methylation changes. One example is 4-(methylnitrosamino)-1-(3-pyridyl)-1-butanone (NNK), a tobacco-specific carcinogen, that is found not only in smoke from traditional cigarettes, but also in E-cig aerosols, albeit at significantly lower levels (Bustamante et al. 2018; Kankanamage et al. 2020). NNK has been reported to alter DNA methylation (Lin et al. 2010; Jin et al. 2015; Taylor et al. 2018; Kankanamage et al. 2020); therefore, E-cig aerosols and their individual constituents should be investigated further for the epigenome altering potential. In the present *in vivo* study, DNA methylation changes that impact cancer pathways were investigated following E-cig exposure in mice. Specifically, this research explored whether subchronic exposure to E-cig aerosols, at relevant concentrations, induces genome wide DNA methylation changes, shedding further light on the molecular mechanisms linking E-cig use to changes in cancer and respiratory pathways.

## Materials and methods

### Generation system and animals

The whole-body inhalation E-cig mouse exposures were performed at the University of California, Davis. E-cig fluid (E-liquid/juice) was carefully prepared to ensure consistency and high quality. A 50:50 mixture of VG and PG was prepared fresh for each use. Nicotine, at a concentration of 24 mg/mL, was added to each E-cig device. The vaping tanks and coils used in this study (KangerTech Ego T2 Clearomizer; KangerTech, Shenzhen, China), were equipped with 1.8 Ω KangerTech T3S coils. The KangerTech Ego T2 was constructed from glass and metal, ensuring durability and precision. Coils were tested using an ohmmeter before each exposure experiment and replaced as needed to maintain consistency and functionality of the system. To generate accurate levels of E-cig aerosols, the KangerTech Ego T2 tanks were connected to an eAerosol E-cig Aerosol Generator (ECAG, e~Aerosols, Central Valley, NY). This specialized, timer-controlled system allowed precise control over puff flow rate, puff duration and interval, and coil wattage. The vaping tanks were operated according to a standardized puff protocol, consisting of 4 s of coil activation at 14 W, followed by 30 s of inactivation, repeated for the entire exposure duration.

These studies compared two strains of male mice, FVBN and C57BL/6, for the effects of E-cig modeled aerosols. For each strain, six male mice were exposed to PG/VG + Nic aerosols, and six male mice were exposed to HEPA-filtered air as controls. Mice were housed in stainless steel cages within stainless steel and glass whole-body inhalation chambers at UC Davis, under the supervision of Dr. Kent Pinkerton. Animals were observed daily throughout the exposure period to assess their general health status, including activity level, grooming behavior, posture, and any signs of respiratory distress. All mice remained active and exhibited normal grooming and behavior patterns throughout the study. No overt clinical abnormalities were observed during the 1-month exposure period. The nicotine groups were exposed to a target aerosol concentration of 150 mg/m^3^, generated from a 50:50 PG/VG blend, with nicotine. Whole-body inhalation exposures were carried out for 3 h/d (5 d/week) for a total of 1 month. Whole-body inhalation exposure was selected to approximate real-world environmental exposure conditions and to minimize restraint-related stress associated with nose-only exposure systems. Chamber aerosol concentrations were continuously monitored to ensure stable and reproducible exposure levels across groups. While chamber concentrations were controlled, individual inhaled dose and deposition fraction were not directly quantified. The cages were rotated through top, middle, and bottom placements within the exposure chamber every two weeks to account for potential concentration variations. The control group was exposed to HEPA-filtered air and rotated in the chamber in the same manner. To assess exposure, mass total suspended particulate (TSP) concentration was measured gravimetrically using Teflon filter samples replaced hourly and was subsequently weighed on a Mettler microbalance in a temperature- and humidity-controlled environment. Particle size distributions were determined using an Intox cascade impactor. The PG/VG + Nic chamber had an average TSP concentration of 157.2 mg/m^3^, with a standard deviation of 26, and a TSP range of 130–209 mg/m^3^. Nicotine analysis for the filtered air chamber was 0.0 mg/m^3^ and 1.24 mg/m^3^ for the PG/VG + Nic chamber. Mice were sacrificed after the last exposure (11–12 weeks of age) by intraperitoneal injection of a lethal dose (175 mg/kg) of pentobarbital sodium. The lungs were removed and snap frozen in liquid nitrogen and then stored at −80 °C for future analysis.

### DNA methylation profiling

#### Sample quality control

Genomic DNA was extracted from tissue samples using the DNeasy Blood & Tissue Kit (QIAGEN^®^ #69506, Venlo, Netherlands), quantified with a NanoDrop spectrophotometer, and 500 ng of each sample was prepared for bisulfite conversion. DNA samples were sent to the NYU Genome Technology Center (NYU Langone Health, New York, NY) and bisulfite converted using the Zymo EZ DNA Methylation Kit (Zymo Research, Irvine, CA) following the manufacturer’s protocol with modifications for compatibility with the Illumina Infinium Mouse Methylation BeadChip (MM285) (Illumina, San Diego, CA). Array BeadChips were scanned on the Illumina iScan system to generate raw intensity IDAT files.

IDAT files were initially evaluated using SeSAMe quality control metrics. Probe- and sample-level quality control was performed using pOOBAH (*p* value with out-of-band probes) and signal detection metrics, and samples or probes failing quality thresholds were removed prior to downstream processing. After filtering, 273 754 CpG loci remained in FVBN mice and 278 837 in C57BL/6 mice. Principal component analysis (PCA) was used to explore data structure, assess potential outliers, and examine major sources of variance (Supplemental Fig 1a and 1b).

#### Methylation value processing

IDAT files were processed using the OpenSesame workflow implemented in the SeSAMe package (Zhou et al. 2018) (Bioconductor: https://bioconductor.org/packages/SeSAMe, GitHub: https://github.com/zwdzwd/sesame) to generate total signal intensity matrices and *β*-values. The Infinium BeadChip uses two bead types per CpG locus, one for methylated (*M*) and one for unmethylated (U) alleles. *β*-values were calculated as *M*/(*M* + *U*), where 0 represents a fully unmethylated site and 1 represents a fully methylated site. *β*-value matrices were used for all subsequent statistical analyses.

#### Differential methylation analysis

Differential methylation was evaluated using the differential methylation locus (DML) function in SeSAMe, which applies mixed linear models to assess associations between DNA methylation levels (*β*-values) and experimental covariates within an EWAS framework (Joubert et al. 2012; Zhou et al. 2018). To ensure both statistical rigor and biological relevance, CpG sites were considered differentially methylated only if they met two criteria:

1. Benjamini–Hochberg FDR ≤ 0.05, and
2. Absolute delta beta (|Δ*β|*) ≥ 0.2, where Δ*β* was defined as the mean *β*-value in PG/VG + Nic-exposed samples minus the mean *β*-value in filtered air controls.

This dual-threshold approach prevents the inclusion of CpGs with statistically significant but biologically negligible effect sizes, which can arise from inherent methylome variability.

#### Downstream analyses

Only CpG sites meeting both FDR and |Δ*β|* thresholds were retained for downstream analyses. Differentially methylated loci (DMLs) were annotated to genes using the Infinium Mouse Methylation Gene Probe Annotation. Gene-level lists derived from these DMLs were used for all pathway enrichment analyses, including ingenuity pathway analysis (IPA), Kyoto Encyclopedia of Genes and Genomes (KEGG), and Gene Ontology (GO). PCA-derived components representing major sources of variance were also evaluated as potential covariates in downstream statistical modeling where appropriate. All analyses were performed using R (Version 2024.12.1) and Bioconductor packages (Gentleman et al. 2004) with support from the Applied Bioinformatics Laboratories (New York, NY) (RRID: SCR_019178).

### Age and epigenetic clock analysis

The SeSAMe package was used to predict DNA methylation age (DNAmAge) in mice by applying the Zhou 347 model (Zhou et al. 2018), which is specifically designed for mouse data. The methylation beta values were extracted from preprocessed MM285 array data, and the predicting function was used in conjunction with the Clock_Zhou347.rds model. This model is based on a set of 347 CpG sites that have been shown to correlate with age in mice. The function predicts the biological age in months by matching the input beta values to the corresponding probes in the model. By using SeSAMe’s processed data and the Zhou 347 model, the biological age of the mice could be accurately estimated, providing insights into their developmental stage based on DNA methylation patterns. An ANOVA was conducted to assess differences in methylation age across strains (FVBN vs. C57BL/6) and treatment groups (filtered air control vs. PG/VG + Nic). Since significant effects were detected, Tukey’s HSD *post hoc* test was performed to evaluate all pairwise comparisons, including strain-specific treatment effects and between-strain differences within each treatment condition. Significance levels were indicated on the plot (*p* < 0.05, *p < 0.01, **p < 0.001).

### IPA and KEGG analysis

#### Ingenuity pathway analysis

After DMLs were annotated to their respective genes, they were analyzed and categorized using IPA (Ingenuity^®^ Systems, www.ingenuity.com). The IPA core analysis tool (Kota et al. 2022; Suthar and Lee 2022) was used to determine the top predicted disease/disorders associated with the hypomethylated and hypermethylated genes observed in both the air control and PG/VG + Nic-exposed C57BL/6 and FVBN male mice.

After DMLs of both strains were annotated to known genes, they were identified using an adjusted *p* value threshold of 0.05 and a fold-change cutoff of |log2 fold change| > 1. A list of genes was compiled, including gene symbols, log2 fold changes, and adjusted *p* values. This gene list was directly used for KEGG pathway and GO enrichment analyses.

#### Kyoto Encyclopedia of Genes and Genomes

For KEGG pathway enrichment analysis, gene lists were analyzed using the enrichKEGG function from the clusterProfilerpackage. Entrez IDs were derived from input gene symbols using the org.Mm.eg.db annotation database, with unmapped genes excluded. The analysis employed a hypergeometric test to assess pathway enrichment, with significance thresholds set at an unadjusted *p* value < 0.05 and a Benjamini–Hochberg-adjusted *q*-value < 0.2. Significantly enriched pathways were visualized using dot plots, highlighting key pathways based on gene ratios and statistical significance.

To ensure accurate statistical inference, the background gene set was restricted to genes represented on the Illumina Mouse Methylation BeadChip (MM285) array. This approach avoids bias introduced by using the full genome as background when only a subset of genes is profiled. The *p* value threshold was selected to balance statistical stringency with biological interpretability, in line with accepted practices in epigenome-wide studies (Maksimovic et al. 2021; Li S et al. 2024; Sahoo and Sundararajan 2024).

#### Gene Ontology

For GO enrichment analysis, the gene lists were analyzed using the enrichGO function, also from the clusterProfiler package. The gene list, including log-fold changes and *p* values, was directly used to assess enrichment. GO significance was defined as a Benjamini–Hochberg-adjusted *p* value < 0.05, and results were summarized using dot plots to display the top enriched terms.

## Results

Differential DNA methylation analysis examines the relationship between the methylation status of individual CpG sites across the genome and phenotypes of interest, conducted on a locus-by-locus basis. For each CpG site, *p* values were calculated using a regression model. Methylation levels in the lungs of adult male mice from both strains were assessed through pairwise comparisons between the PG/VG + Nic group and filtered air controls (*n* = 6 per treatment group). Genome-wide DNA methylation array analysis of lung tissue (*n* = 6 male mice per treatment group/per strain) collected from PG/VG + Nic exposed mice identified 12 000 significant E-cig-associated CpG sites differentially methylated in the C57BL/6 strain (false discovery rate, FDR < 0.05); 34 442 such sites were identified in the FVBN strain (*p* < 0.05) (Figures 1 and 2, Table 1). Using the SeSAMe pipeline, hypermethylated CpG sites were defined as those with a delta beta (Δ*β*) 0.2 and a Benjamini–Hochberg FDR-adjusted *p* value < 0.05. Hypomethylated CpG sites were defined as those with a delta beta (Δ*β*) ≤ −0.2 and a Benjamini–Hochberg FDR-adjusted *p* value < 0.05. Differential methylation results were visualized using volcano plots, with Δβ on the *x*-axis and − log10 (*p* value) on the *y*-axis. In the C57BL/6 strain, 5158 (43%) of the significant CpG sites were hypermethylated, while 6842 (57.1%) were hypomethylated. In comparison, 15 908 (46.2%) of the significant CpG sites were hypermethylated in the FVBN strain, and 18 534 (53.8%) were hypomethylated in the same mouse strain (Table 1).

Among these differentially methylated CpG sites, 3973 (33.1%) were assigned to known genes in the C57BL/6 strain, and 12 260 (35.6%) were annotated to known genes in the FVBN strain using the Illumina methylation array’s annotation library. Critically, the Illumina platform quantified methylation at individual CpG sites; gene associations were assigned *post hoc* based on genomic proximity or regulatory context. In the C57BL/6 male mice, 2300 (57.9%) of the 3973 differentially methylated genes in the PG/VG + Nic treatment group were significantly hypomethylated compared to the filtered air controls (*p* < 0.05) (Table 1). The PG/VG + Nic exposed FVBN male mice revealed 6731 (54.9%) significantly hypomethylated genes (Table 1), with 1120 shared hypomethylated genes between the two strains (Table 1).

The PG/VG + Nic exposed C57BL/6 male mice exhibited 1673 (42.1%) significantly hypermethylated genes compared to filtered air controls (Table 1); in comparison, the FVBN male mice exposed 5529 (45.1%) hypermethylated genes (Table 1). Of these, 688 hypermethylated genes were shared between both mouse strains (Table 1). Among the 3973 significantly methylated CpG sites annotated for known genes in the C57BL/6 mouse strain, 544 (13.6%) were in regulatory gene promoter regions, while the majority were located in transcription start site-associated gene bodies (TSS_BODY). Similarly, in the FVBN strain, only 1371 of 12 260 (11.2%) differentially methylated genes had promoter-associated CpGs, with the majority also found in TSS_BODY regions.

The beta density is a mathematical model for proportions or probabilities characterized by shape parameters alpha and beta and is well-suited for describing DNA methylation levels (beta values), which range from 0 to 1 (Qi et al. 2022). The shape of the distribution curve can vary—uniform, unimodal, or bimodal—depending on alpha or beta shape. The beta value distributions of both C57BL/6 and FVBN in this study displayed two peaks, which are similar to the human methylome profile (Pidsley et al. 2016) and align with other *in vivo* studies (Garcia-Prieto et al. 2022) (Figures 3 and 4). Both strains displayed the expected bimodal *β*-value distribution typical of mammalian methylomes.

A Manhattan plot was generated to visualize the significant CpG sites for both mouse strains (Figures 5 and 6). The results highlight distinct patterns of genetic associations between the two strains, with notable differences in the distribution and density of associations across chromosomes. In the FVBN strain, the highest number of significant CpG associations was observed on chromosomes 10 (1456), 11 (2002), and 17 (1275); chromosomes 12 (1030), 13 (1104), and 15 (1224). Chromosome 18 (824) also showed substantial numbers of associations, suggesting widespread genetic contributions across the genome. In contrast, chromosomes 2, 3, 4, 5, 6, 7, and 9 exhibited fewer CpG associations than observed for the other chromosomes, with counts ranging from 1 to 6 (Figure 5).

For the C57BL/6 strain, the distribution of significant CpG associations with the various chromosomes was generally lower in number compared to FVBN but followed a similar pattern. The highest CpG counts were observed on chromosomes 10 (463), 11 (623), and 17 (402); chromosomes 12 (347), 13 (391), 14 (350), and 15 (345) also showed moderate levels of CpG associations. Notably, chromosomes 2, 3, 4, and 7 had very few CpG associations, with counts of two or fewer (Figure 6). These data suggest that while both mouse strains share some common regions of genetic interest (e.g. chromosomes 10, 11, and 17), the FVBN strain exhibits a higher overall density of CpG associations. The sparse CpG associations observed on mouse chromosomes 2–9 are not related to the experimental exposures, but primarily stem from technical constraints of the Illumina MM285 array platform, which selectively targets regulatory regions like promoters and CpG islands while excluding repetitive or structurally complex genomic areas (Bibikova et al. 2011; Pidsley et al. 2016; Zhou et al. 2022). Conversely, the concentration of significant associations on chromosomes 10, 11, and 17 corresponds to evolutionarily conserved regions of the mouse genome compared to those in humans.

These genome regions contain critical immune and cancer-related loci, including the MHC complex involved in antigen presentation, HOX genes regulating cellular differentiation, and T-cell receptor genes essential for adaptive immunity (Saunders and Seldin 1990; Cruz-Tapias et al. 2013; Hubert and Wellik 2023). Thus, the differential methylation landscape not only reflects platform-specific technical biases but also reveals functionally important epigenetic conservation between mouse and human genomes, particularly in pathways relevant to oncogenesis and immune surveillance.

### Gene enrichment associated with E-cigarette exposure: a comprehensive analysis

#### IPA analysis reveals disease pathways affected by PG/VG + Nic exposure in C57BL/6 and FVBN mice

Ingenuity pathway analysis was conducted to identify the top predicted diseases and disorders linked to the hypo- and hypermethylated genes observed in PG/VG + Nic exposed male C57BL/6 and FVBN mice. The results highlight the top five predicted diseases/disorders associated with altered gene expressions in both mouse strains. The top 5 predicted diseases identified (considering both hypo- and hypermethylated genes) were similar across both strains. Expanding the IPA output beyond the top five categories did not reveal any additional biologically distinct pathways, as lower-ranked predictions largely reflected overlapping gene sets or mechanistic sub-classifications within these same broad disease domains; therefore, presentation of the top five categories captures the dominant biological processes affected while avoiding redundancy. These broad predictions included cancer, organismal injury and abnormalities, endocrine system disorders, gastrointestinal, and neurological diseases. However, as mentioned earlier, the number of identified ‘genes’ associated with differentially methylated CpG sites for each strain was quantitatively different. For example, the hypermethylated gene set in the C57BL/6 strain included 1031 genes associated with a cancer pathway, whereas the FVBN strain had 2422 genes linked to the same pathway (Tables 2 and 3). This is not unexpected given that the absolute number of differentially methylated genes (12 260) in the FVBN strain (the more sensitive strain compared to C57BL/6) was more than double the number identified in C57BL/6 mice (3976). However, when compared to the total number of differentially methylated genes in each strain, the percentage of identified genes with altered expression associated with cancer pathways was relatively similar, 25.9% in C57BL/6 versus 19.8% in FVBN.

#### KEGG enrichment of cancer-related pathways in hypermethylated and hypomethylated gene sets of PG/VG + Nic-exposed C57BL/6 mice

Using KEGG analysis methods, for the C57BL/6 mice exposed to E-cig aerosols, distinct differences were observed between the hypermethylated and hypomethylated gene sets. KEGG pathway analysis of the hypermethylated gene set revealed enrichment in several cancer pathways, including calcium signaling, cyclic adenosine monophosphate (cAMP) signaling, Wnt/β-catenin (Wnt) signaling, retrograde endocannabinoid signaling, glutamatergic synapse, adherens junction, colorectal cancer, circadian entrainment, and gonadotropin-releasing hormone (GnRH) signaling (Figure 7, Table 4).

In contrast to the hypermethylated gene set, the hypomethylated gene set was enriched in KEGG analysis for signaling pathways, including those related to the cytoskeleton in muscle cells, axon guidance, mechanistic targeting of rapamycin (mTOR), AMP-activated protein kinase (AMPK), T-helper 17 (Th17) cell differentiation, T-cell receptor signaling, and the sphingolipid signaling pathway. Additionally, pathways associated with cardiomyopathies—such as arrhythmogenic right ventricular cardiomyopathy, hypertrophic cardiomyopathy, and dilated cardiomyopathy—were also enriched (Figure 8, Table 4). Taken together, mTOR, AMPK, Th17, T-cell receptor signaling, Wnt signaling, and colorectal cancer are just some of the pathways that were enriched and vital for immune regulation, energy signaling, and cardiovascular health.

#### KEGG enrichment of cancer-related pathways in hypermethylated and hypomethylated gene sets of PG/VG + Nic-exposed FVBN mice

For FVBN mice exposed to PG/VG + Nic, KEGG analysis revealed that the hypermethylated gene set compared to filtered air-exposed mice was enriched for signaling pathways, including calcium signaling, cytoskeleton in muscle cells, Wnt, Hippo, growth hormone synthesis, thyroid hormone, GnRH, long-term potentiation, tyrosine metabolism, and beta-alanine metabolism (Figure 9, Table 5). Wnt, Hippo, calcium signaling, beta-alanine metabolism, and adherens junction are crucial for maintaining cellular structure, metabolic regulation, and proper functioning of neuronal and hormonal systems (Bootman and Bultynck 2020; Wu et al. 2024). Notably, Wnt and Hippo signaling pathways are well-known for their roles in cell proliferation, differentiation, and tissue homeostasis, and any dysregulation of any of these pathways is frequently implicated in cancer development and progression (Sethi and Vidal-Puig 2010; Fu et al. 2022; Zhao et al. 2022).

In contrast to the hypermethylated gene set, the hypomethylated gene set in FVBN mice was enriched for different pathways, including mitogen-activated protein kinases (MAPKs), Wnt, Ras-associated protein-1 (Rap1), proteoglycans associated with cancer in general, breast cancer, phospholipase D, regulation of pluripotent stem cells, dilated cardiomyopathy, and circadian entrainment (Figure 10, Table 5); MAPK, Rap1, Wnt are definitively linked to cancer due to their critical roles in regulating cell proliferation, survival, differentiation, and metastasis (He et al. 2023; Zhang et al. 2023; Tripathi et al. 2024).

KEGG pathway analysis of hypermethylated gene sets in C57BL/6 and FVBN mice revealed that both strains shared enrichment in calcium signaling and Wnt signaling pathways, highlighting a consistent disruption in these key biological processes. However, FVBN mice also exhibited hypermethylation in pathways linked to growth hormone, thyroid hormone regulation, and neuronal processes, highlighting strain-specific effects related to hormonal regulation and neuronal function. While the hypomethylated gene sets revealed that C57BL/6 mice demonstrated enrichment pathways related to immune function, energy regulation, and cardiomyopathies, FVBN mice exhibited hypomethylation of pathways associated with cancer progression, stem cell regulation, and cell signaling.

#### GO analysis of cancer-related pathways in hypermethylated and hypomethylated gene sets of PG/VG + Nic-exposed C57BL/6 mice

Gene Ontology is a comprehensive bioinformatics initiative that provides a structured vocabulary for annotating gene functions across different species. GO offers a hierarchical and species-independent framework for interpreting gene roles in biological systems. Unlike pathway-focused resources such as KEGG, which maps detailed biochemical interactions, or IPA, which centers on disease and therapeutic contexts, GO serves as a descriptive, universal system for classifying gene function. This broader scope allows GO to complement more applied tools by offering a standardized foundation for understanding how genes contribute to diverse cellular and physiological processes. In the context of epigenetic regulation, GO enrichment analysis is particularly useful for revealing high-level biological themes associated with altered gene expression or methylation.

In this study, PG/VG + Nic exposed C57BL/6 mice revealed the hypermethylated gene set to be enriched for biological processes associated with regulation of membrane potential, cell junction assembly, mitotic cell cycle phase transition, muscle system processes, positive regulation of the cell cycle, cognition, learning or memory, cell cycle G1/S phase transition, and protein localization to cell junctions (Figure 11, Table 6). The observed hypomethylated gene set in C57BL/6 mice was enriched for biological processes related to negative regulation of protein modification processes, transmembrane receptor protein serine/threonine kinase signaling pathways, negative regulation of phosphorus and phosphate metabolic processes, negative regulation of phosphorylation and protein phosphorylation, dendrite development, embryonic organ morphogenesis, dephosphorylation, and regulation of cell morphogenesis (Figure 12, Table 6). For example, calcium signaling influences membrane potential and muscle processes, while Wnt signaling controls cell cycle progression and cell junction assembly (Díaz-Piña ~ et al. 2024; Su et al. 2024). These connections highlight how KEGG pathways provide the molecular mechanisms underlying the broader biological processes defined by GO. For example, while GO terms like ‘cell junction assembly’ or ‘regulation of membrane potential’ describe high-level cellular functions, KEGG pathways such as Wnt or calcium signaling detail the specific gene interactions that drive these processes. Thus, GO offers the functional context, while KEGG reveals the mechanistic basis, together offering a more integrated view of gene function.

#### GO analysis of cancer-related pathways in hypermethylated and hypomethylated gene sets of PG/VG + Nic-exposed FVBN mice

In FVBN mice exposed to PG/VG + Nic, the hypermethylated gene set revealed disruptions in biological processes linked to gland development, regulation of developmental growth, and neurogenesis. All of which are essential for maintaining normal cell differentiation, tissue development, and growth regulation (Figure 13, Table 7).

The hypomethylated gene set in FVBN mice was enriched for biological processes associated with lymphocyte differentiation, cell junction assembly, epithelial tube morphogenesis, dendrite development, small GTPase-mediated signal transduction, regulation of developmental growth, cell–substrate adhesion, regulation of GTPase activity, embryonic organ morphogenesis, and locomotory behavior (Figure 14, Table 7.).

Epithelial tube morphogenesis and embryonic organ morphogenesis are involved in tissue and organ formation, while small GTPase-mediated signal transduction, regulation of GTPase activity, cell–substrate adhesion, and locomotory behavior are all involved with cellular signaling and movement. The previously mentioned biological processes act to ensure proper growth, structural integrity, and functional coordination in multicellular organisms (Ashburner et al. 2000; Lewis et al. 2019).

### Strain-specific effects of inhalated E-cigarette aerosols on epigenetic clock

DNA methylation alterations were further examined using an epigenetic clock (Clock_Zhou347) (Zhou et al. 2018). The epigenetic clock is a computational model that estimates biological age based on DNA methylation patterns at specific CpG sites. Using the predictAge tool from the SeSAMe package, the biological age of the samples was determined. The analysis revealed significant strain-dependent effects on biological aging (full factorial ANOVA: *F*(3,20) = 152.20, *p* = 6.15 × 10^–14^). While PG/VG + Nic exposed FVBN mice showed significant epigenetic age acceleration compared to filtered air control (median increase from 4.17 to 4.68 months; *p* = 0.024), C57BL/6 mice exhibited no treatment-induced effect (9.36 vs. 9.59 months; *p* = 0.674) on the same parameter. Significant differences in aging between strains were observed in both treatment groups: filtered air exposed FVBN had an epigenetic age of 4.17 months versus that observed in C57BL/6, i.e. 9.36 months (*p* = 1.76 × 10^−7^). There was also a significant difference between both strains in the PG/VG + Nic exposure groups, with FVBN revealed an epigenetic age of 4.68 months, C57 mice showed an age of 9.59 months (*p* = 1.29 × 10^−9^) (Figure 15).

## Discussion

Emerging evidence from both mouse and human studies highlights a potential association between vaping and epigenetic alterations that can influence cancer development (Choi and Lee 2013; Huynh et al. 2020; Xie et al. 2021; Shehata et al. 2023). Recent research has revealed both overlapping and distinct epigenetic changes when comparing traditional cigarette smokers and E-cig users to nonsmokers. This study investigated the effects of inhaled E-cig aerosol exposure on genomic DNA methylation changes in the lungs of two strains of male mice, C57BL/6 and FVBN, compared to non-vaping controls. Results revealed alterations in methylation patterns, many of which are associated with cancer-related pathways. Moreover, these investigations demonstrate that exposure to aerosols of PG and VG in a 50:50 ratio with added nicotine (24 mg/mL; PG/VG + Nic) induces significant changes in CpG methylation across the genome, with over 3000 significant CpG sites identified in C57BL/6 mice and over 12 000 in FVBN mice. Our studies further demonstrated that most of the CpGs annotated to known genes were located within gene bodies.

Although cigarette smoke and E-cig aerosols share components, such as nicotine, heavy metals, and VOCs, they differ in that E-cig aerosols lack the combustion byproducts found in traditional cigarette smoke. As a result, E-cigs could contribute to epigenetic changes in the genome that differ from those attributed to smoking. The absence of combustion products in E-cig aerosols has been shown to lessen the number and/or concentration of some harmful constituents compared to traditional cigarette smoking (Lampos et al. 2019; Balfour et al. 2021; Aherrera et al. 2023). However, the presence of nicotine and other aerosolized chemicals in E-cigs appears to be more than sufficient to disrupt epigenetic regulation, particularly in pathways critical for cancer development (Hikisz and Jacenik 2023; Shehata et al. 2023).

In this study, KEGG analysis reveals that inhaled E-cig aerosols with nicotine leads to hypermethylation in the Wnt signaling pathway. The Wnt pathway is crucial for cell proliferation, differentiation, and migration, which are processes involved in embryonic development, cellular division, and cancer progression. Hypermethylation of this particular pathway has been associated with tumorigenesis, as it can disrupt normal cellular functions and interact with other cancer-associated pathways, such as Hippo/YAP (Sharma et al. 2021; Zhao et al. 2022; Zhang et al. 2023). In support of our results, hypermethylation of Wnt was also seen in a study investigating molecular pathways in cultured oral cells of E-cig vapers and traditional cigarette smokers. E-cig users who provided epithelial cells for this *in vitro* study were defined as those who reported current use of E-cigs at least three times a week for a minimum of 6 months (Merecz-Sadowska et al. 2020). Ingenuity pathway analysis of these cultured cells revealed the Wnt signaling pathway at the top of the canonical pathway in E-cig users (*p* = 4.7 × 10^−5^), while the integrin signaling pathway (*p* = 1.42 × 10^−8^) was the most significantly altered in traditional cigarette smokers. This study highlights the relevance of oral epithelial cells in understanding the cancer risks of both E-cig and traditional cigarette use, as over 90% of cancers are of epithelial origin. Interestingly, components of the Wnt signaling pathway appeared in both hypermethylated and hypomethylated gene sets, particularly in FVBN mice. As this study focused on genome-wide methylation profiling, transcriptional validation was not performed. However, integrating gene-specific transcriptomic analyses in future work will clarify which Wnt components were functionally altered by E-cig aerosol exposure. The observed E-cig-induced hypermethylation in the Wnt signaling pathway in cultured oral cells from vapers supports our similar findings in lung tissue, further reinforcing the potential cancer risks of E-cig use (Tommasi et al. 2019).

In addition to the Wnt pathway, other cancer-related pathways were altered in the current study, including those involved in colorectal cancer, breast cancer, and cell signaling cascades, including MAPK, Hippo, and mTOR pathways (Sever and Brugge 2015; Sanchez-Vega et al. 2018; Patil et al. 2021; Zhao et al. 2022; Li Q et al. 2024). Notably, hypomethylation was observed in the MAPK and AMPK pathways. AMPK interacts with MAPK signaling to modulate stress responses and influence cell proliferation, migration, and survival in cancer. Their crosstalk underscores cooperative roles in maintaining metabolic and oncogenic balance within tumor cells (Keerthana et al. 2023). While no studies (to our knowledge) have specifically examined the effects of inhaled E-cig aerosol exposure on AMPK pathways specifically, research has been conducted on the effects of traditional cigarette smoking on this same signaling pathway. In one large-scale meta-analysis, blood samples from 17 675 individuals across 26 independent studies were analyzed for smoking-related DNA methylation changes. The study found that genes associated with both AMPK and MAPK pathways were significantly enriched and overexpressed. Among the 57 pathways linked to various types of cancer (FDR < 0.05), AMPK and MAPK signaling were strongly implicated in cigarette smoking-related carcinogenic processes (Ma and Li 2017).

In our *in vivo* mouse study, we observed hypermethylation of Rab26, a gene involved in MAPK pathway regulation, in E-cig aerosol-exposed FVBN mice. This epigenetic alteration of Rab26 in lung tissue suggests potential disruption of MAPK-mediated inflammatory control following E-cig aerosol exposure. Supporting mechanistic evidence for our findings comes from an *in vitro* study in which cultured BEAS-2B cells exposed to cigarette smoke condensate (CSE) exhibited ROS-mediated methylation of Rab26, with functional consequences on airway inflammation (He et al. 2023). In that model, overexpression of Rab26 attenuated the inflammatory response by partially inactivating p38 and c-Jun N-terminal kinases (JNKs), whereas silencing Rab26 exacerbated inflammation. The hypermethylation of Rab26 observed in our E-cig exposed FVBN mice, combined with the mechanistic BEAS-2B findings, suggests that inhaled aerosols, including those from E-cigs, may promote airway inflammation by reducing Rab26 activity and altering downstream MAPK signaling (Wang K et al. 2022; He et al. 2023). Although this proposed mechanism has not been directly tested *in vivo*, the results indicate a possible shared epigenetic pathway between traditional cigarette smoke and E-cig aerosols.

While our results reveal enrichment of multiple cancer-related signaling pathways (e.g. Wnt, MAPK, Hippo, and mTOR), these findings do not establish a direct tumor formation or cancer onset. Instead, they indicate that E-cig aerosol exposure may disrupt epigenetic regulation within pathways commonly implicated in carcinogenesis. Therefore, while E-cig exposure could contribute to cellular and molecular conditions that favor cancer development, additional chronic exposure and mechanistic *in vivo* and *in vitro* studies are required to determine causality.

Subsequent to the KEGG pathway enrichment analysis, the biological implications of the differential DNA methylation patterns using GO were explored in our study. Results from this study also demonstrated hypermethylation of biological processes associated with cell cycle regulation, including the G1/S phase transition, positive regulation of the cell cycle, and mitotic cell cycle phase transition. These biological processes are crucial for controlling cell division and proliferation, and their disruption could potentially lead to uncontrolled cell growth, a hallmark of cancer (Cui et al. 2015; Han et al. 2020; He et al. 2023; Nakata et al. 2024; Ding et al. 2025). Additionally, hypermethylation in biological processes related to membrane potential and cell junction assembly could potentially impair cellular communication and tissue organization, further contributing to tumorigenesis (Almagro et al. 2022; de la Calle-Fabregat et al. 2024; Wang L-J et al. 2024; Zhang et al. 2024). Similarly, the hypomethylated gene set observed in our study was enriched for biological processes involved in protein modification and phosphorylation, both processes critical for regulating cell growth, survival, and apoptosis.

Disruption of these hypomethylated pathways could lead to aberrant signaling that supports tumorigenesis by enabling cells to bypass normal growth control mechanisms (Zong et al. 2019; Das et al. 2021; Babar et al. 2022). Other enriched biological processes in our analyses were linked to developmental growth and neurogenesis, processes that are tightly regulated during normal tissue development, further indicating potential dysregulation relevant to cancer (González-Castrillón et al. 2023). Taken together, our findings provide evidence that exposure to nicotine-containing E-cig aerosols induces epigenetic changes in cancer-related pathways that could ultimately contribute to cancer development or worsen cancer prognosis.

Another emerging marker of cancer risk is DNAmAge, a biomarker that reflects cumulative epigenetic changes associated with aging and disease progression (Horvath and Raj 2018; Hao et al. 2023). Previous evidence suggests that DNAmAge is closely linked to an increased risk of death from natural causes and could also serve as a predictor of life expectancy (Horvath 2013; Horvath and Raj 2018; Levine et al. 2018; Kresovich et al. 2019; Hao et al. 2023). Recent studies have highlighted the impact of cigarette smoking and E-cig use on epigenetic aging and cancer risk (Cardenas et al. 2022; Fang et al. 2023; Herzog et al. 2024). One epidemiological study evaluated cigarette smoking-associated DNA methylation signatures in a cohort from the Costa Rican Study on Longevity and Healthy Aging (CRELES), including participants from the high longevity region of Nicoya (Cardenas et al. 2022). Herein, former smokers exhibited significant epigenetic age acceleration, with increases in multiple age acceleration clocks, alongside shorter telomere length. Additionally, no evidence of effect modification was observed within the high longevity region (Cardenas et al. 2022). A more recent epidemiological study (Song et al. 2023) explored the effects of traditional cigarette smoking and vaping on epigenetic aging; the results of that study demonstrated that both smokers and E-cig users exhibited older biological age, compared to nonsmokers. The authors of this study linked accelerated aging to immune-related signaling pathways and cytokine production, also known to be involved in cancer progression. The results from the aforementioned epidemiological studies suggest that exposure to E-cig aerosols could share similar biological effects with cigarette smoking, accelerating aging and increasing the risk of cancer and other chronic illnesses. Based on this, our laboratory explored the strain-specific influences of E-cig exposure on DNAmAge. In our study, methylation age analysis revealed a notable increase in FVBN mice exposed to PG/VG + Nic aerosols compared to air controls. In contrast, despite significant observed E-cig-induced DNA methylation alterations, the C57BL/6 mice showed minimal changes in epigenetic aging, highlighting the complexity of methylation changes in aging and cancer susceptibility. These results suggest that the epigenetic aging response to E-cig aerosol exposure varies by genetic background and may reflect differences in susceptibility to aging-related or cancer-associated epigenetic effects. The observed variability underscores the importance of considering genetic factors when evaluating the long-term health consequences of vaping, particularly in the context of biological aging and cancer risk.

Lastly, the strain-specific differences in DNA methylation patterns observed in this study provide critical insights into how genetic background can influence susceptibility to cancer following E-cig exposure, echoing patterns previously observed in mice exposed to traditional cigarette smoke (Nadziejko et al. 2007; Gordon and Bosland 2009). Overall, FVBN mice exhibited a greater number of hypermethylated and hypomethylated genes following E-cig exposure, with notable enrichment in pathways related to cell differentiation, stem cell regulation, and hormonal signaling, all of which are intricately linked to cancer development. In contrast, exposure of male C57BL/6 mice revealed significant E-cig exposure enrichment of hypomethylation in immune regulation and cardiovascular pathways, suggesting distinct biological vulnerabilities less directly associated with cancer risk. Overall, the results from our study point to potential mechanisms by which exposure to E-cig aerosols could elevate the risk of certain cancers and other chronic diseases, particularly in genetically predisposed populations (Tommasi et al. 2019; Molony et al. 2023; Shehata et al. 2023). Although FVBN mice exhibited a greater number of DMLs compared to C57BL/6 mice, both strains were exposed under identical whole-body inhalation conditions to E-cig aerosols with equivalent chamber aerosol concentrations. Respiratory parameters such as tidal volume and breathing frequency were not directly measured; however, the mice were age-matched adult males, minimizing substantial physiological variability. While subtle strain-dependent differences in aerosol deposition cannot be entirely excluded, the observed methylation differences are more likely attributable to intrinsic genetic and/or epigenetic differences rather than differential aerosol exposure. These strain-specific differences further underscore the importance of tailoring experimental models of vaping to reflect genetic variability, enabling more precise and targeted research designs.

### Limitations

This study provides valuable insights into the impact of E-cig exposure on genome-wide DNA methylation and its potential links to cancer-related pathways. However, there are several recognizable limitations in our study. This study focused exclusively on adult male mice to minimize hormonal variability and allow strain-specific comparisons. Moreover, data demonstrate a higher prevalence of vaping in males. Further research, including both males and females, is desperately needed to determine any sex-specific differences in toxicological and epigenetic responses to E-cig aerosol exposure.

Additionally, our study focused on E-cigs containing a mixture of 50:50 PG/VG with nicotine, but due to time- and financial constraints, did not examine the effects of PG/VG alone, which could have helped clarify the specific role of nicotine. Nicotine may directly influence DNA methylation through receptor-mediated signaling and oxidative stress, whereas PG/VG can thermally degrade into reactive carbonyl compounds such as formaldehyde and acrolein that may also affect the epigenome (Aherrera et al. 2023). Thus, the methylation changes observed in this study likely result from both thermal degradation products and nicotine-driven molecular signaling, warranting additional PG/VG-only and nicotine-free exposure models to isolate these effects. Future investigations should aim to disentangle the independent and combined effects of these components. Additionally, animals were exposed using a whole-body inhalation system to reduce stress associated with nose-only platforms and, as much as possible, better approximate real-world environmental exposure conditions; however, individual inhaled dose and deposition fraction were not directly quantified. Although chamber aerosol concentrations were carefully controlled and monitored, variability in respiratory parameters between animals cannot be entirely excluded. While animals were monitored daily for general health and exhibited no overt clinical abnormalities during the exposure period, body weights were not recorded. Therefore, quantitative assessment of potential systemic toxicity was limited.

The nicotine concentration used in this study (24 mg/mL), while reflective of nicotine levels commercially available at the commencement of this study, has dramatically increased over time. Current nicotine levels available for sale can reach up to 60 mg/mL, warranting additional investigation at higher nicotine concentrations. While the use of a lower nicotine concentration could be viewed as a limitation, it demonstrates that relatively low levels of nicotine can bring about alterations in the epigenome. Finally, longer-term, chronic E-cig exposure studies could have better reflected human use, and temporal and longitudinal evaluations post-exposure could be advantageous for a better understanding of any delayed/or persistent effects of E-cig use. Furthermore, although pathway enrichment analyses identified networks commonly implicated in cancer biology, this study only evaluated DNA methylation changes and did not assess tumor formation or functional gene expression. Therefore, the findings herein should be interpreted as evidence of epigenetic pathway perturbation of genome pathways associated with cancer, rather than as definitive proof of carcinogenesis.

## Conclusions

In summary, the results from our studies demonstrate that vaping nicotine-containing E-cigs (at a relatively low concentration of nicotine) produces extensive epigenetic changes, particularly via alterations in key cancer-related pathways, including those involved in cell cycle regulation, signaling, and development. These results suggest that E-cig use could pose potential risks for public health, particularly to the extreme users (i.e. adolescents and young adults), contributing to cancer risk by disrupting cellular processes critical to tumorigenesis. Importantly, while enrichment analyses identified pathways commonly implicated in cancer biology, this study evaluated DNA methylation alterations and did not assess tumor formation, functional gene expression, or disease outcomes. Therefore, the observed epigenetic changes should be interpreted as potential molecular events rather than direct evidence of carcinogenesis. Furthermore, the current study highlights the complexity of E-cig’s impact on DNA methylation across different genetic backgrounds, underscoring the need for further research into the long-term health consequences of E-cig exposure on different populations. Although the global market for E-cigs is still small compared to tobacco cigarettes, it is growing very rapidly, particularly among adolescents. Thus, based on the prevalence of use throughout the world and the current research data, more E-cig studies are desperately needed to decipher chronic diseases in future public health and regulatory strategies.

## Supplementary Material

Supp 1

## Figures and Tables

**Figure 1.**
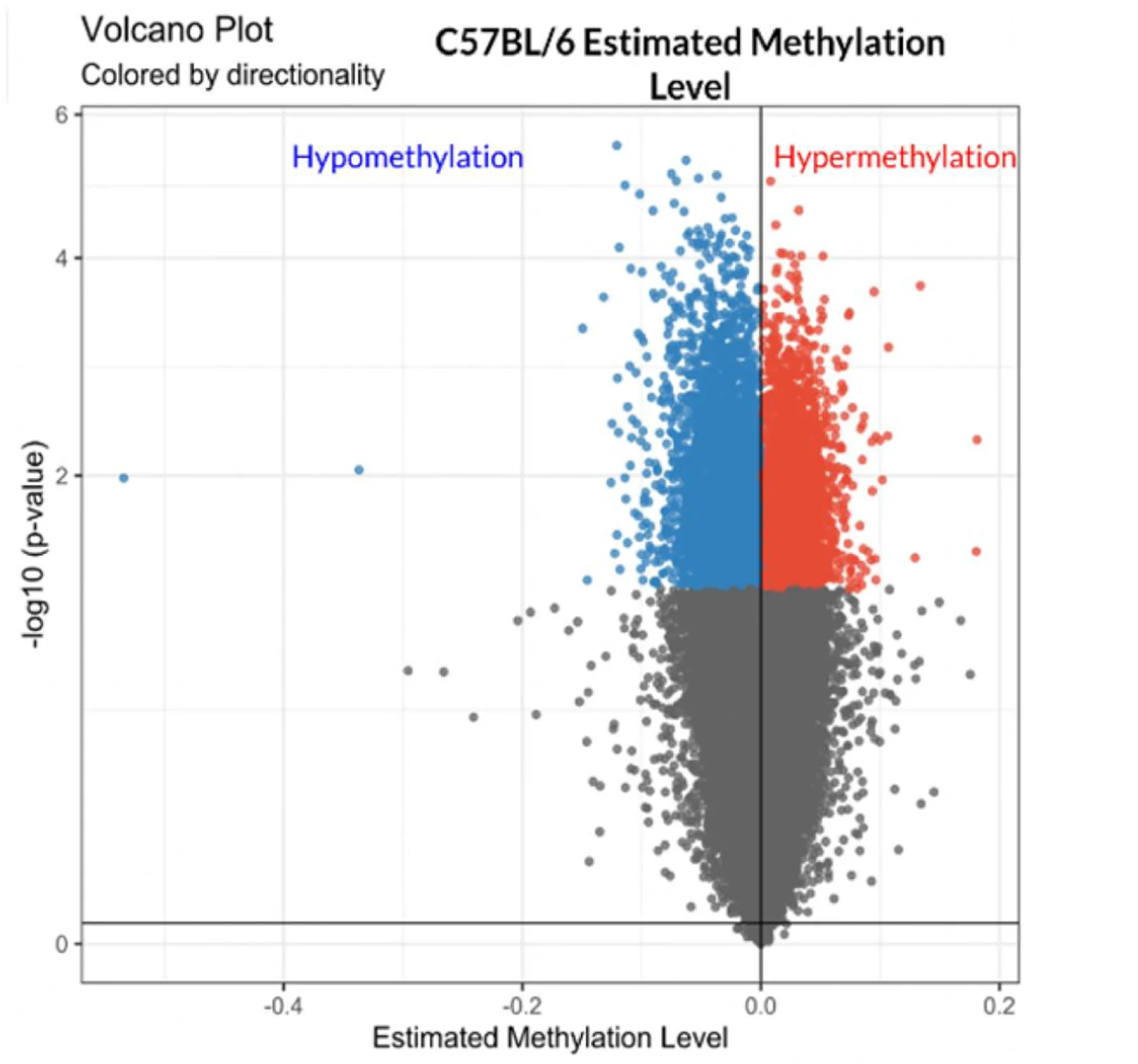
Volcano plot of−log10 (*p* value) against delta beta values representing the methylation differences between PG/VG + Nic- and filtered air-exposed C57BL/6 mice. Non-significant differentially methylated loci are in grey. Significant (false discovery rate (FDR) = 5%) differentially methylated loci are shown in blue and red circles. Hypermethylated CpG sites were defined as having a delta beta (Δ*β*) ≥ 0.2 and a Benjamini–Hochberg FDR-adjusted *p* value <0.05 and are shown in red circles. Hypomethylated CpG sites were defined as having a delta beta (Δ*β*) ≤ −0.2 and a Benjamini–Hochberg FDR-adjusted *p* value < 0.05, blue circles. Differential methylation was visualized using volcano plots, with Δ*β* on the *x*-axis and−log10 (*p* value) on the *y*-axis.

**Figure 2.**
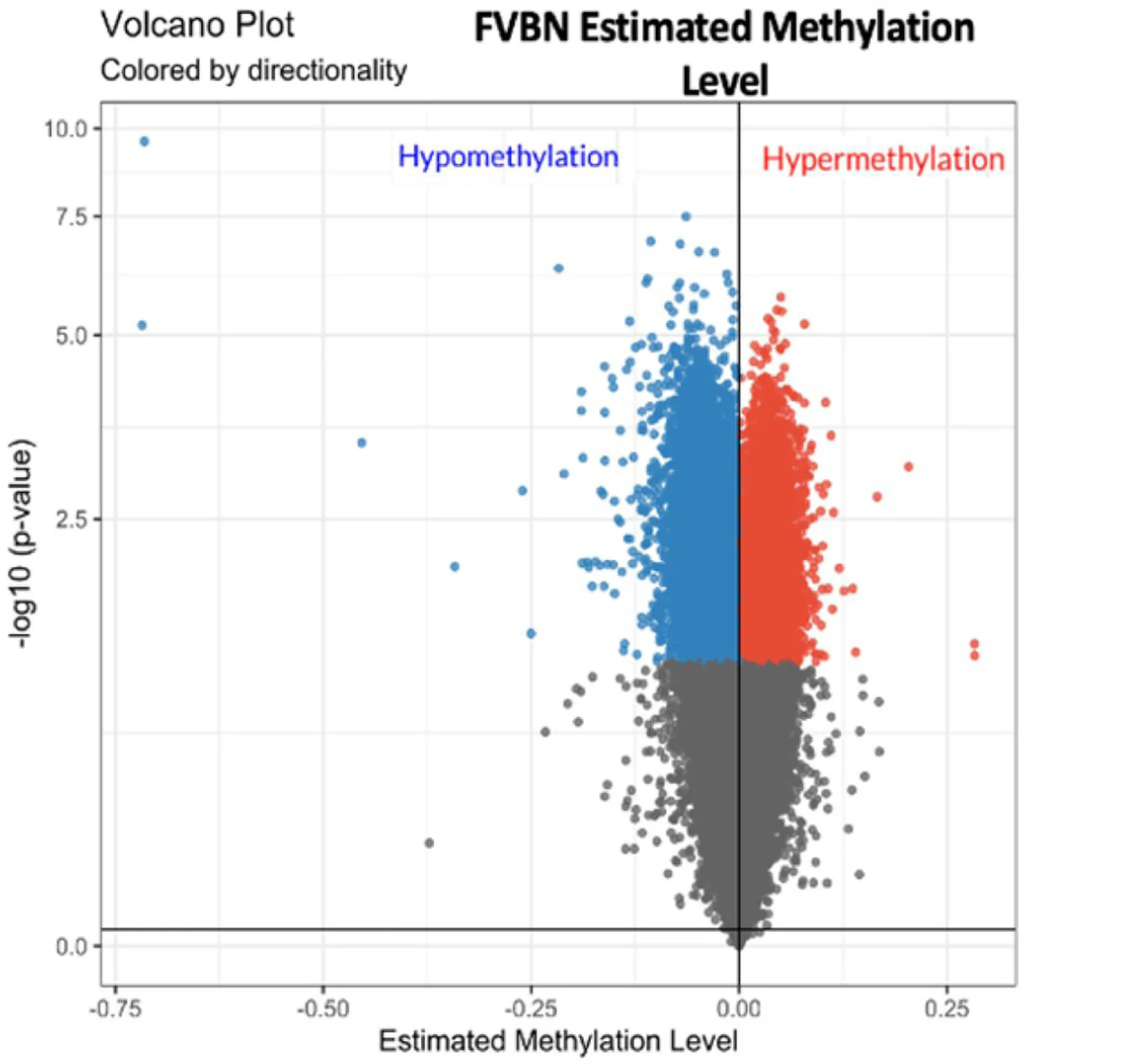
Volcano plot of−log10 (*p* value) against delta beta value representing the methylation differences between PG/VG + Nic and filtered air-exposed FVBN mice. Non-significant differentially methylated loci are in grey. Significant (false discovery rate (FDR) = 5%) differentially methylated loci are shown in blue and red circles. Hypermethylated CpG sites were defined as having a delta beta (Δ*β*) ≥ 0.2 and a Benjamini–Hochberg FDR-adjusted *p* value < 0.05 are shown in red circles. Hypomethylated CpG sites were defined as having a delta beta (Δ*β*) ≤ −0.2 and a Benjamini–Hochberg FDR-adjusted *p* value < 0.05, blue circles. Differential methylation was visualized using volcano plots, with Δ*β* on the *x*-axis and − log10 (*p* value) on the *y*-axis.

**Figure 3.**
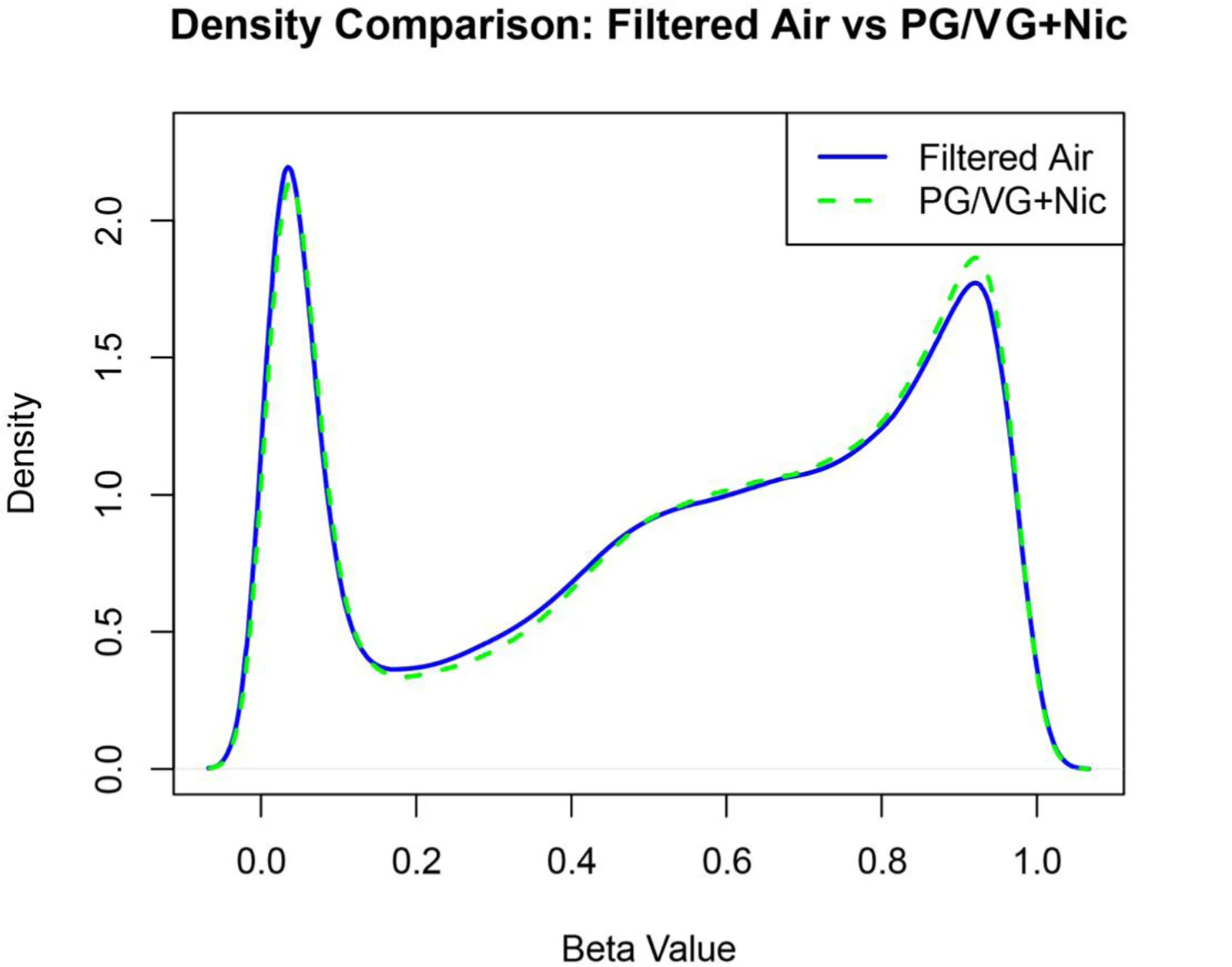
Distribution of beta values in C57BL/6.

**Figure 4.**
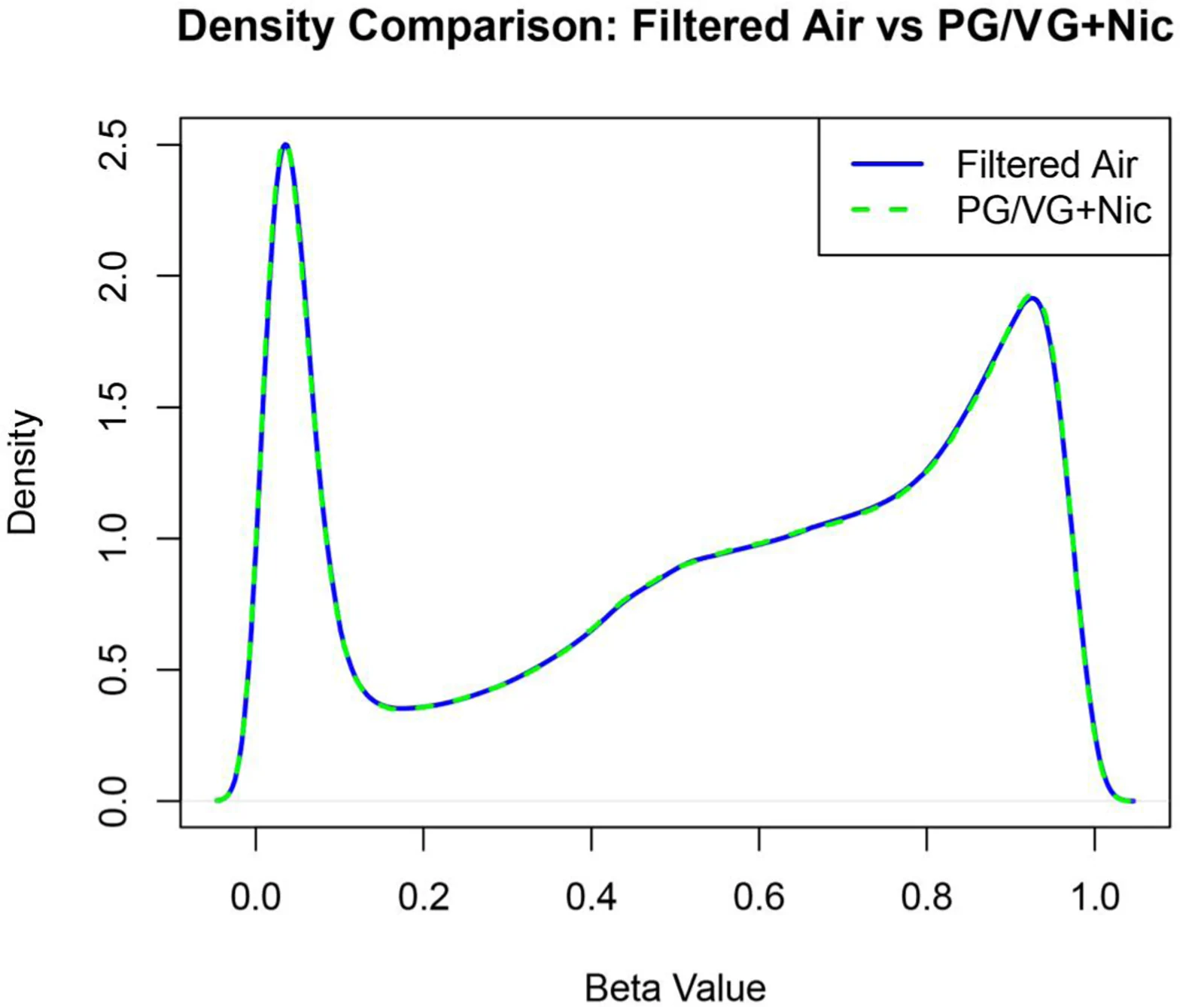
Distribution of beta values in FVBN.

**Figure 5.**
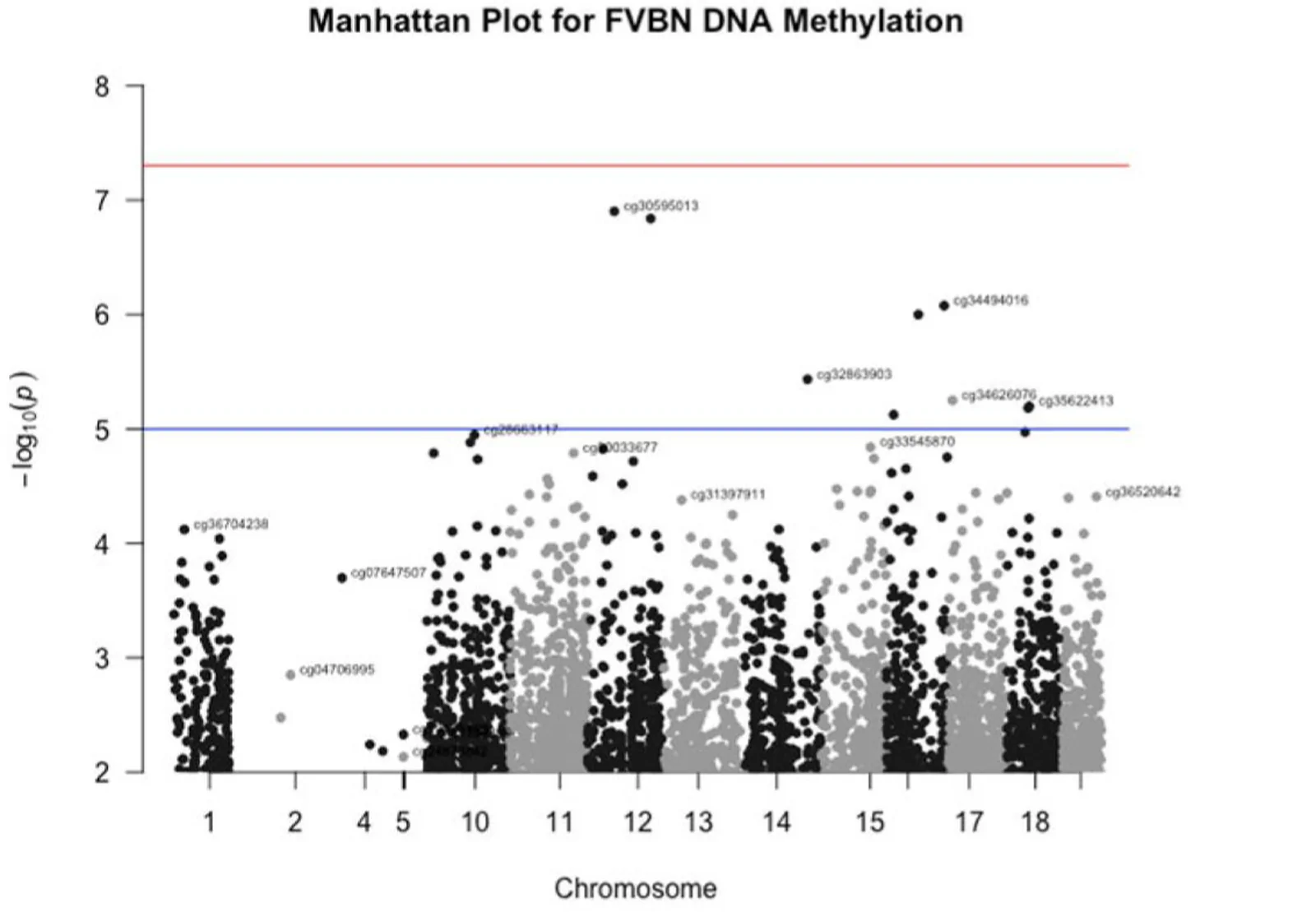
Manhattan plot displays CpG methylation levels across the genome in FVBN mice, with each point representing a CpG site. The *x*-axis represents genomic location, and the *y*-axis indicates methylation levels (−log10 (*p* value)). Peaks highlight regions of significant or variable methylation. The blue line (5) indicates the suggestive significance threshold (*p* = 1 × 10^−5^), and the red line (7.5) marks the genome-wide significance threshold (*p* = 5 × 10^−8^).

**Figure 6.**
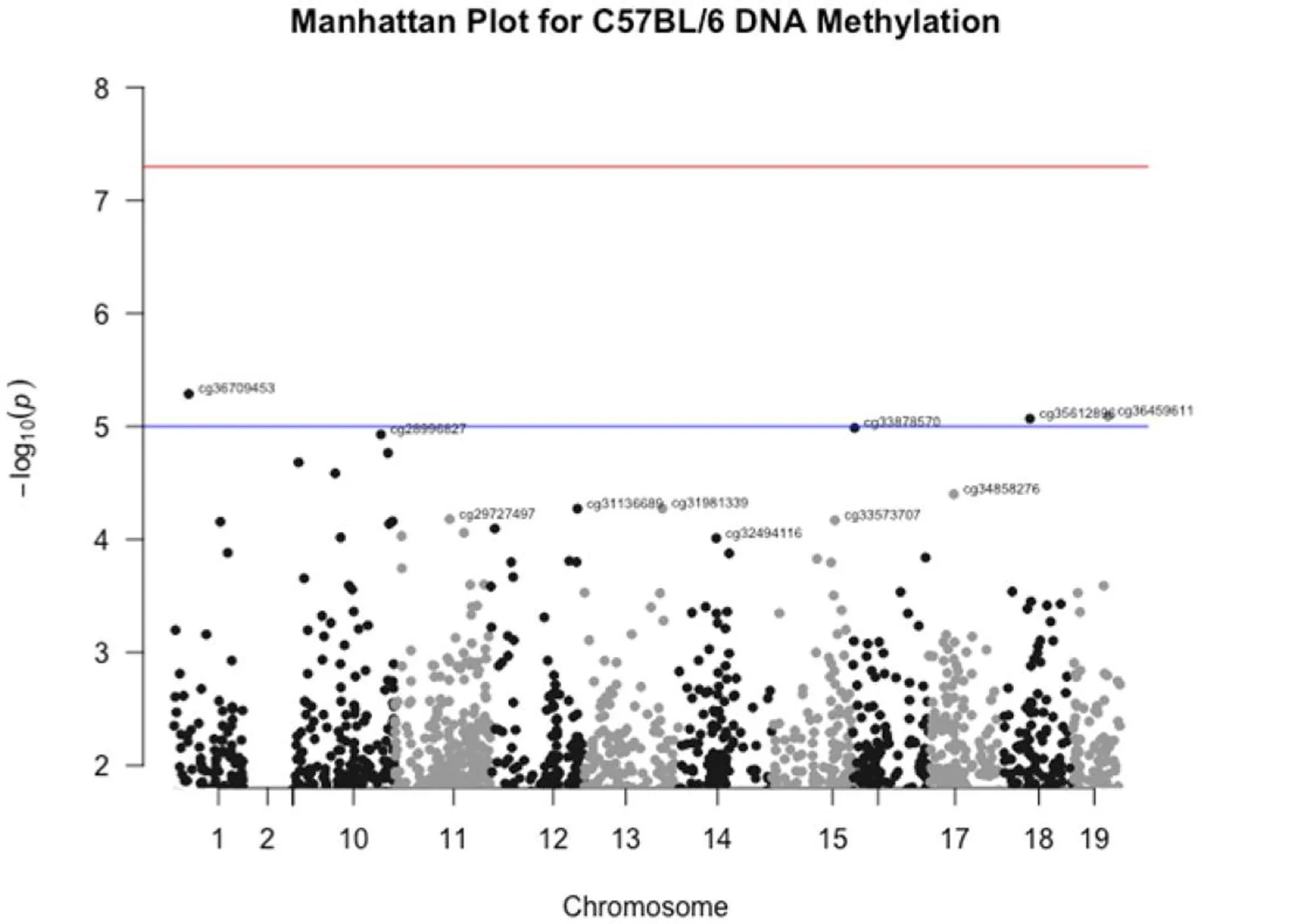
Manhattan plot displays CpG methylation levels across the genome in C57BL/6 mice, with each point representing a CpG site. The *x*-axis represents genomic location, and the *y*-axis indicates methylation levels (−log10 (*p* values)). Peaks highlight regions of significant or variable methylation. The blue line (5) indicates the suggestive significance threshold (*p* = 1 × 10^−5^), and the red line (7.5) marks the genome-wide significance threshold (*p* = 5 × 10^−8^).

**Figure 7.**
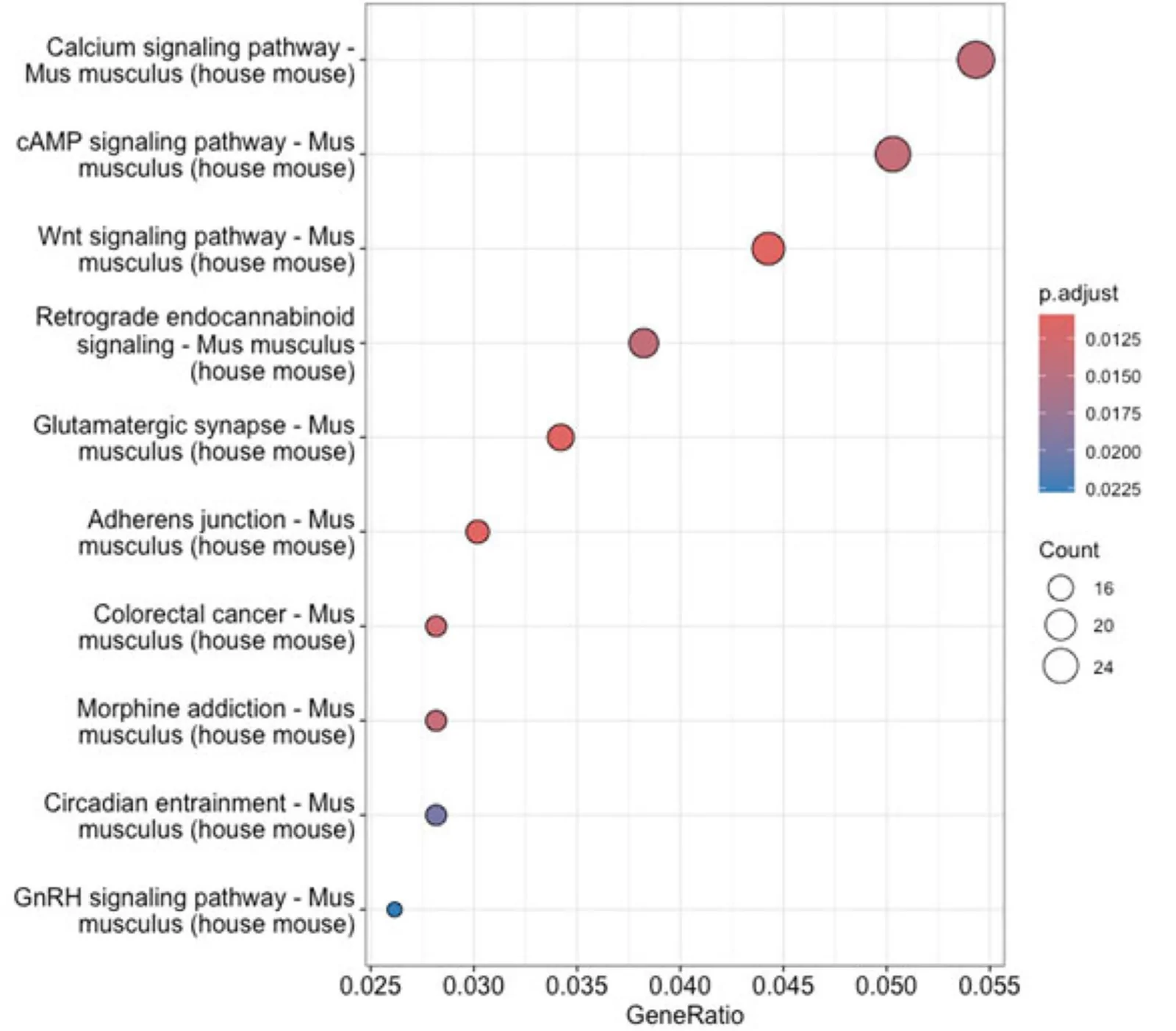
Dot plot of KEGG pathway enrichment analysis for hypermethylated gene sets in C57BL/6 mice. Dot size represents the number of genes reflected, and the color indicates the significance level (−log10 (*p* value)). The *x*-axis shows the gene ratio (number of significant genes/total genes in the pathway). Top 10 enriched pathways are labeled, highlighting biological processes associated with increased DNA methylation in each strain.

**Figure 8.**
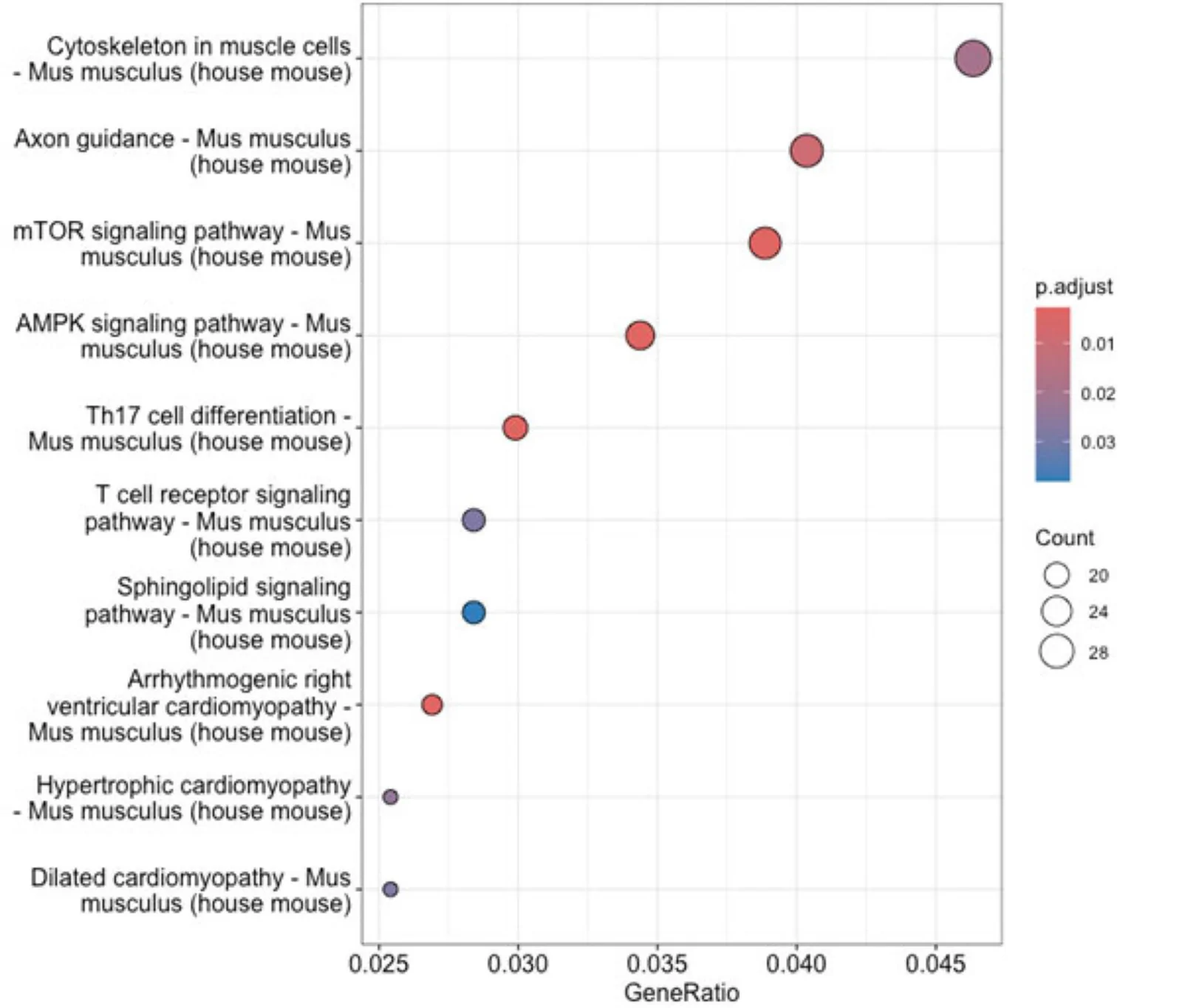
Dot plot of KEGG pathway enrichment analysis for hypomethylated gene sets in C57BL/6 mice. Dot size represents the number of genes reflected, and color indicates the significance level (−log10 (*p* value)). The *x*-axis shows the gene ratio (number of significant genes/total genes in the pathway). Top 10 enriched pathways are labeled, highlighting biological processes associated with increased methylation in each mouse strain.

**Figure 9.**
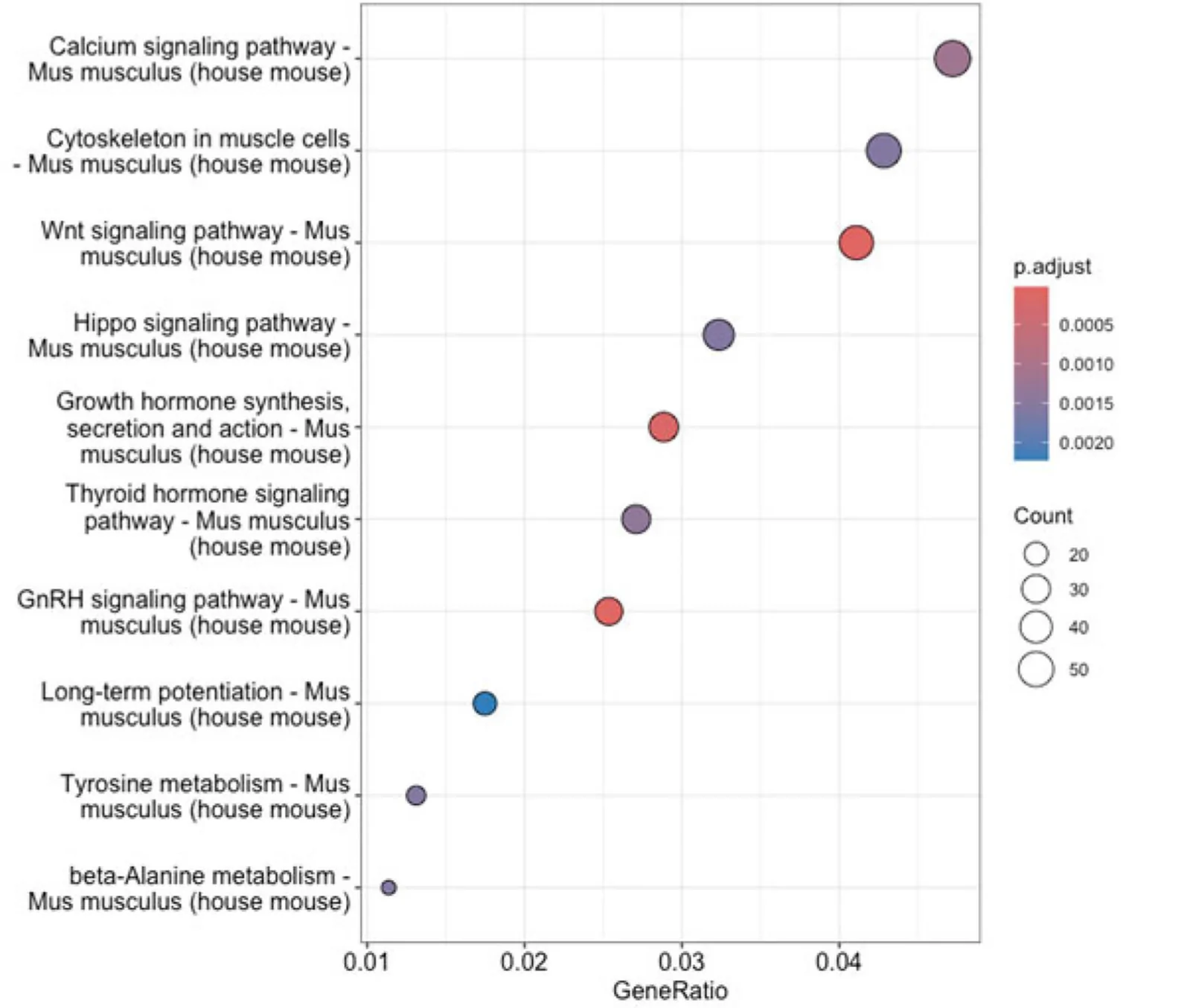
Dot plot of KEGG pathway enrichment analysis for hypermethylated gene sets in FVBN mice. Dot size represents the number of genes, and color indicates the significance level (−log10 (*p* value)). The *x*-axis represents the gene ratio (number of significant genes/total genes in the pathway). Top 10 enriched pathways are labeled, highlighting biological processes associated with increased methylation in each mouse strain.

**Figure 10.**
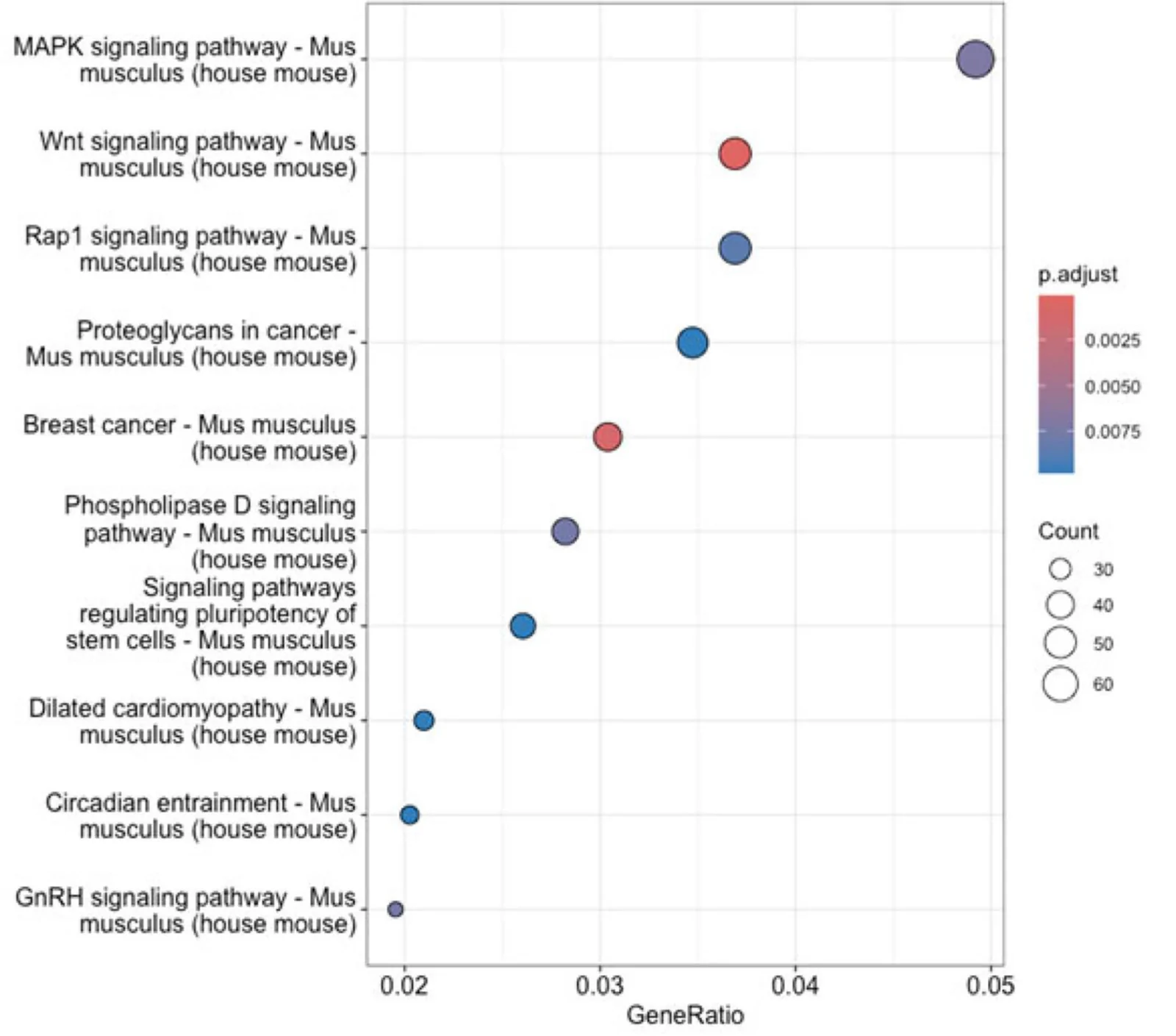
Dot plot of KEGG pathway enrichment analysis for hypomethylated gene sets in FVBN mice. Dot size represents the number of genes, and color indicates the significance level (log10 (*p* value)). The *x*-axis represents the gene ratio (number of significant genes/total genes in the pathway). Top 10 enriched pathways are labeled, highlighting biological processes associated with increased methylation in each strain.

**Figure 11.**
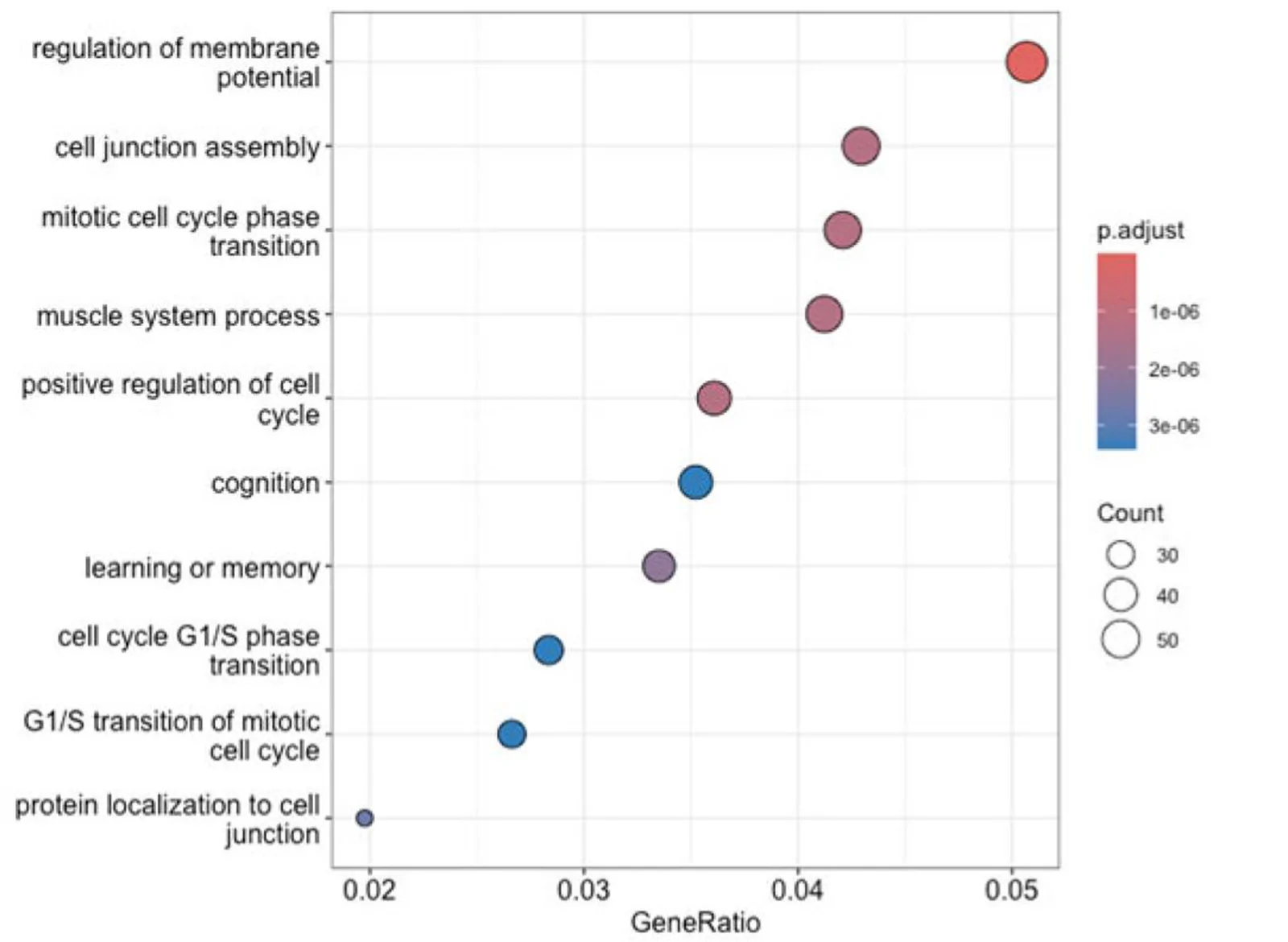
Dot plot of GO pathway enrichment analysis for hypermethylated gene sets in C57BL/6 mice. Dot size represents the number of genes, and color indicates the significance level (−log10 (*p* value)). The *x*-axis represents the gene ratio (number of significant genes/total genes in the pathway). Top 10 enriched pathways are labeled, highlighting biological processes associated with increased methylation in each mouse strain.

**Figure 12.**
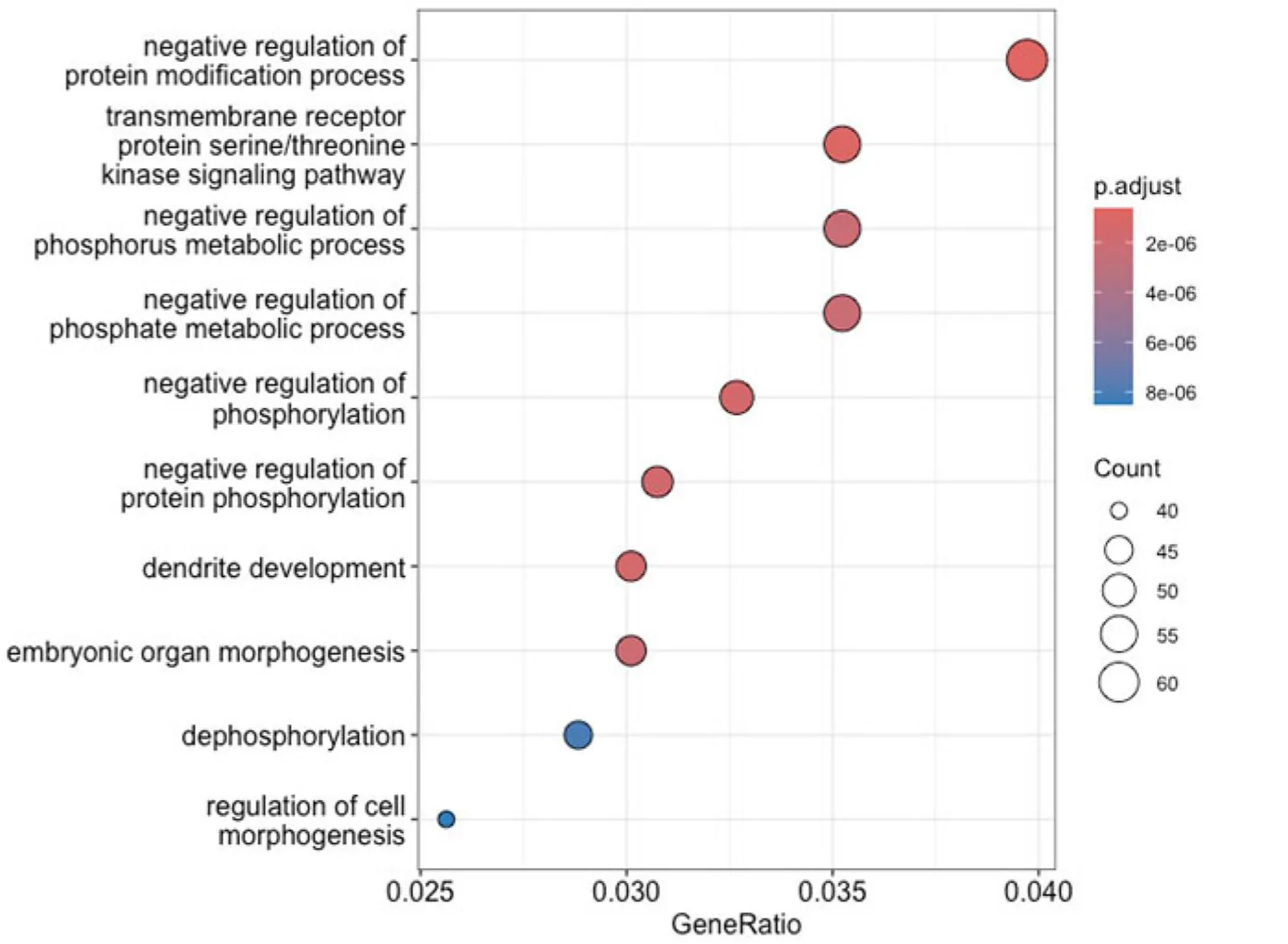
Dot plot of GO pathway enrichment analysis for hypomethylated gene sets in C57BL/6 mice. Dot size represents the number of genes, and color indicates the significance level (−log10 (*p* value)). The *x*-axis shows the gene ratio (number of significant genes/total genes in the pathway). Top 10 enriched pathways are labeled, highlighting biological processes associated with increased methylation in each strain.

**Figure 13.**
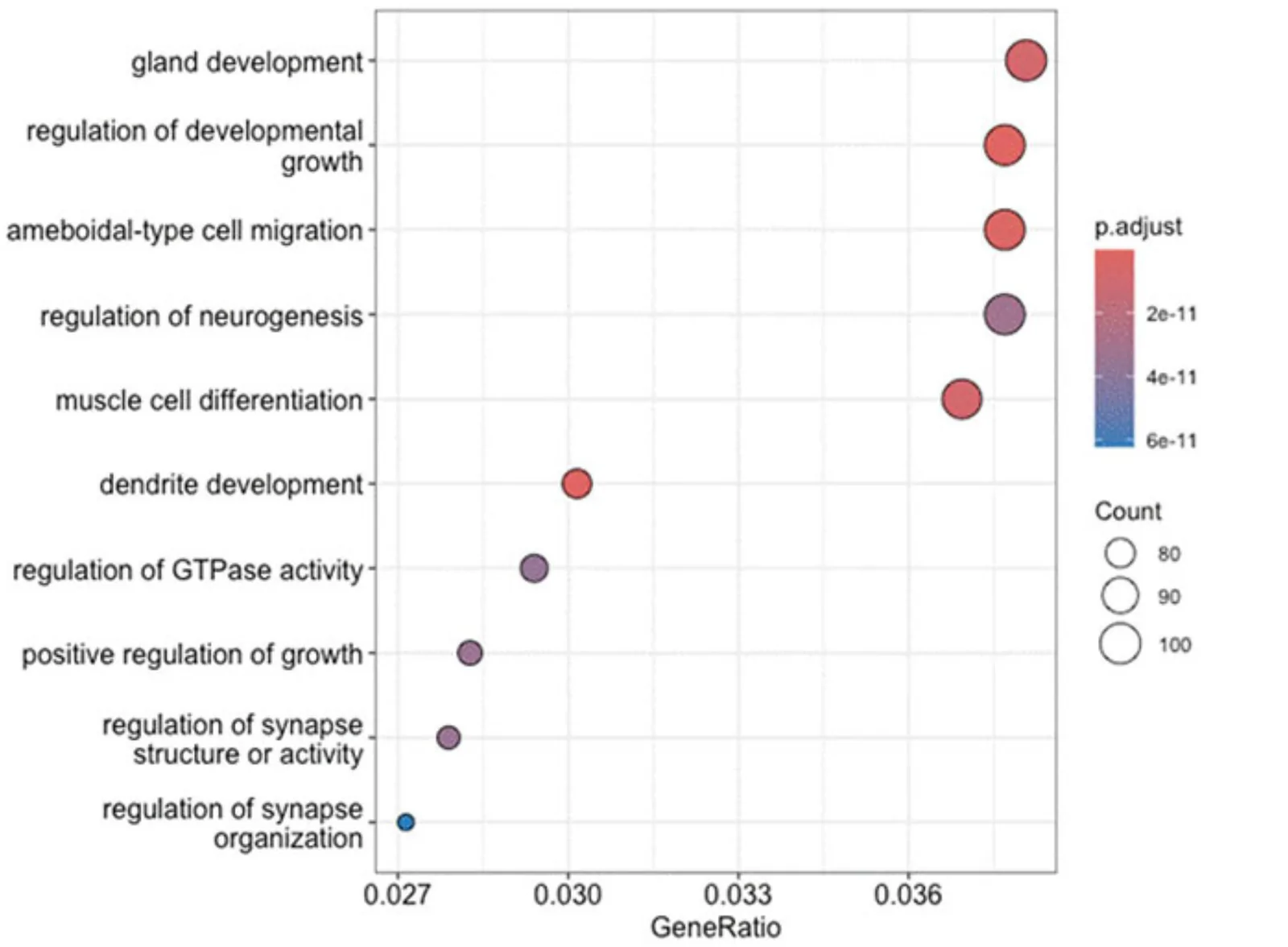
Dot plot of GO pathway enrichment analysis for hypermethylated gene sets in FVBN mice. Dot size represents the number of genes, and color indicates the significance level (−log10 (*p* value)). The *x*-axis shows the gene ratio (number of significant genes/total genes in the pathway). Top 10 enriched pathways are labeled, highlighting biological processes associated with increased methylation in each strain.

**Figure 14.**
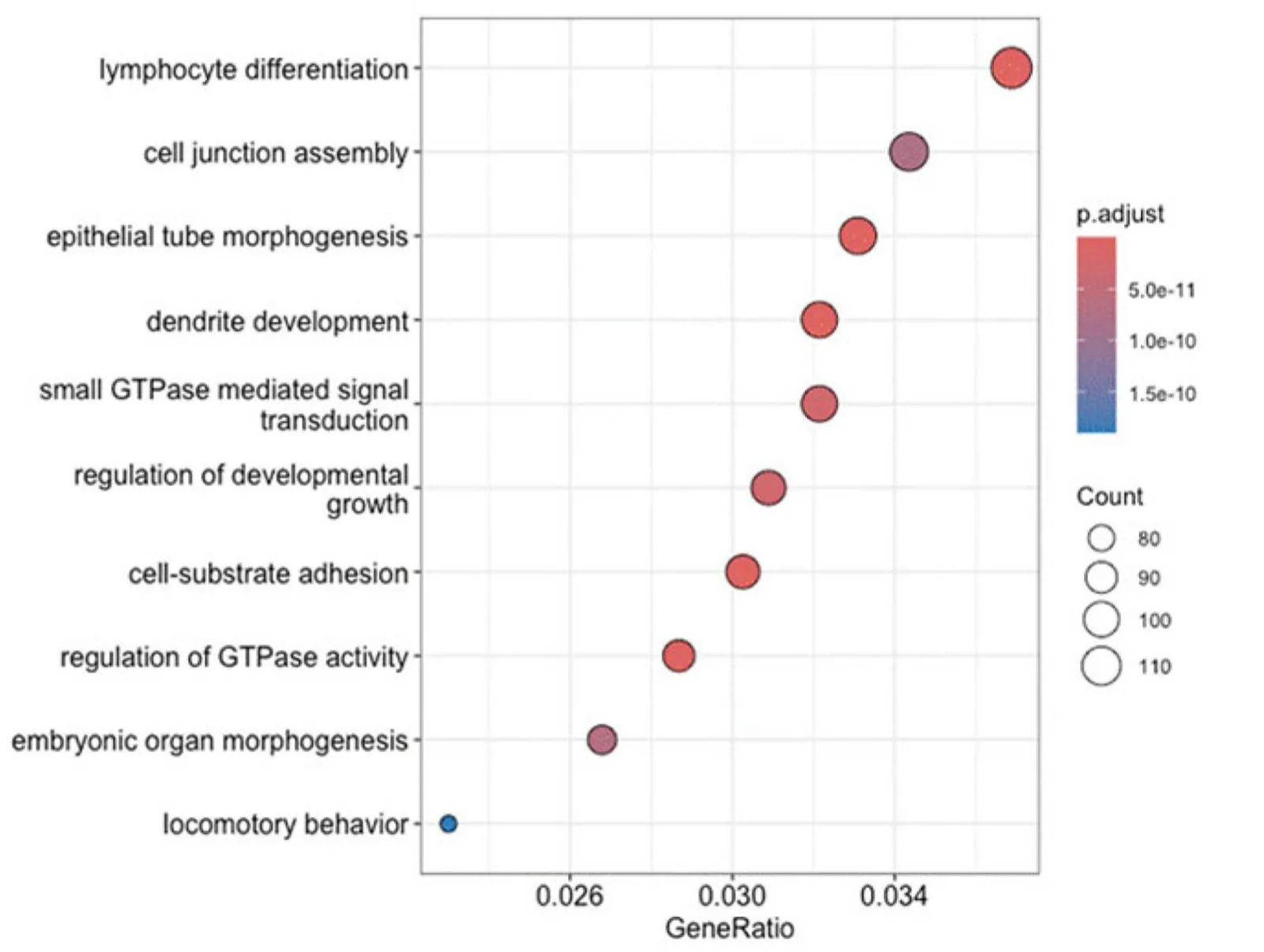
Dot plot of GO pathway enrichment analysis for hypomethylated gene sets in FVBN mice. Dot size represents the number of genes, and color indicates the significance level (−log10 (*p* value)). The *x* axis shows the gene ratio (number of significant genes/total genes in the pathway). The top 10 enriched pathways are labeled, highlighting biological processes associated with increased methylation in each mouse strain.

**Figure 15.**
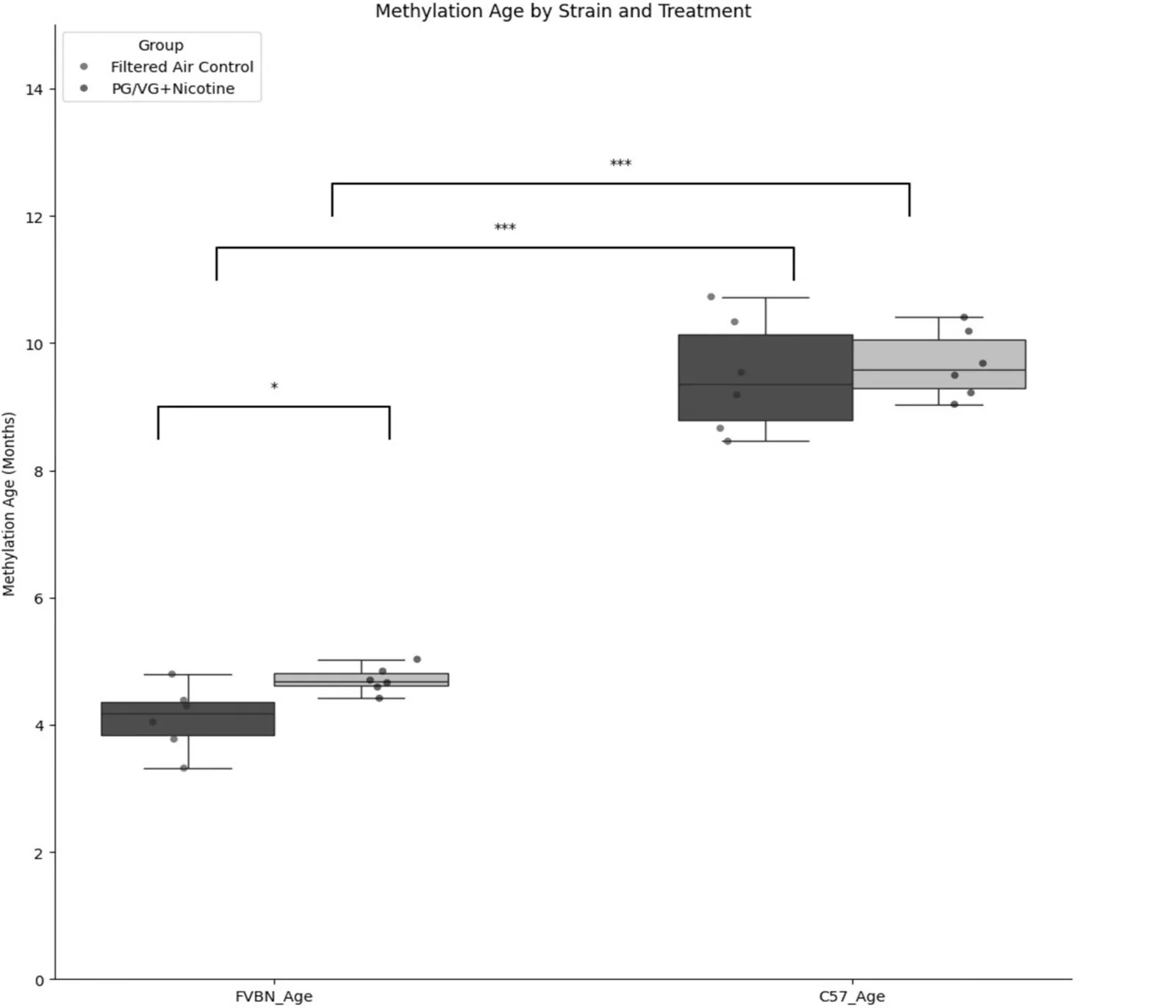
Strain- and treatment-specific effects on DNA methylation age. Boxplots show methylation age (months) in FVBN, and C57BL/6 mice exposed to filtered air (gray) or PG/VG + Nic (light gray). ANOVA revealed significant treatment effects in FVBN (*F*(1,10) = 7.08, *p* = 0.024) but not C57BL/6 mice (*F*(1,10) = 0.19, *p* = 0.674). Discernable strain differences were also observed between both filtered air (*F*(1,10) = 160.29, *p* = 1.8 × 10^−7^) and PG/VG + Nic conditions (*F*(1,10) = 444.19, *p* = 1.3 × 10^−9^). Asterisks indicate significant post hoc Tukey HSD comparisons (*p < 0.05, ***p < 0.001). Individual data points are overlaid.

**Table 1.** Significantly hyper- and hypomethylated CpGs.

Mouse strain	Significantly hypermethylated CpGs	Significantly hypermethylated annotated genes	Significantly hypomethylated CpGs	Significantly hypomethylated annotated genes

C57BL/6	5158 (43%)	1673 (42.1 %)	6842 (57.1%)	2300 (57.9%)
FVBN	15 908 (46.2%)	5529 (45.1%)	18 534 (53.8%)	6731 (54.9%)

Significantly annotated genes; *p* value < 0.05.

**Table 2.** Top 5 IPA disease/disorder categories for significantly hypermethylated genes for both C57BL/6 and FVBN male E-cig exposed mice compared to filtered-air controls.

Diseases and Disorders		
Name C57BL/6	p-value range	# Molecules

**Cancer**	5.46E-06 – 4.46E-74	1414
**Organismal Injury and Abnormalities**	5.46E-06 – 4.46E-74	1452
**Endocrine System Disorders**	3.28E-06 – 5.19E-74	1306
**Gastrointestinal Disease**	3.17E-06– 2.21E-60	1320
**Neurological Disease**	2.09E-06 – 9.23E-50	1076
Name FVBN	p-value range	# Molecules

**Cancer**	3.08E-11 – 2.27E-137	2919
**Organismal Injury and Abnormalities**	3.08E-11 – 2.27E-137	2984
**Endocrine System Disorders**	3.08E-11 – 1.37E-119	2617
**Gastrointestinal Disease**	2.78E-11 – 1.71E-113	2711
**Neurological Disease**	2.52E-11 – 8.54E-93	2214

The number of molecules, calculated from the gene expression data, indicates how many molecules/genes in the disease/disorder pathway were altered. The *p* value range indicates the range of significance for the # molecules involved in each pathway.

**Table 3.** Top 5 IPA disease and disorder categories for significantly hypomethylated genes of both C57BL/6 and FVBN male exposed mice, compared to filtered-air control.

Diseases and Disorders		
Name C57BL/6	p-value range	# Molecules

**Cancer**	2.10E-06 – 4.39E-48	1031
**Gastrointestinal Disease**	1.81E-06 – 4.39E-48	975
**Organismal Injury and Abnormalities**	2.13E-06 – 4.39E-48	1060
**Dermatological Diseases and Conditions**	1.69E-06 – 1.33E-44	807
**Reproductive System Disease**	4.28E-07 – 1.31E-37	790
Name FVBN	p-value range	# Molecules

**Cancer**	1.49E-10 – 5.21E-111	2422
**Organismal Injury and Abnormalities**	1.64E-10 – 5.21 E-111	2478
**Gastrointestinal Disease**	1.02E-11 – 1.50E-103	2275
**Endocrine System Disorders**	2.06E-11 – 1.35E-100	2204
**Neurological Disease**	1.64E-10 – 6.89E-90	1904

The number of molecules, calculated from the gene expression data, indicates how many molecules/genes in the disease/disorder pathway were altered. The *p* value range indicates the range of significance for the molecules involved in the pathway.

**Table 4.** KEGG pathway enrichment analysis of differentially hypermethylated and hypomethylated genes in the C57BL/6 strain.

C57BL/6 KEGG analysis of hypermethylated genes	C57BL/6 KEGG analysis of hypomethylated genes

**Calcium signaling pathway**	Cytoskeleton in muscle cells
**cAMP signaling pathway**	Axon guidance
**Wnt signaling pathway**	mTOR signaling pathway
**Retrograde endocannabinoid signaling**	AMPK signaling pathway
**Glutamatergic synapse**	Th17 cell differentiation
**Adherens junction**	T cell receptor signaling pathway
**Colorectal cancer**	Sphingolipid signaling pathway
**Morphine addiction**	Arrhythmogenic right ventricular cardiomyopathy
**Circadian entrainment**	Hypertrophic cardiomyopathy
**GnRH signaling pathway**	Dilated cardiomyopathy

The table lists enriched pathways, and pathways related to cancer are highlighted in bold.

**Table 5.** KEGG pathway enrichment analysis of differentially hypermethylated and hypomethylated genes in the FVBN strain.

FVBN KEGG analysis of hypermethylated genes	FVBN KEGG analysis of hypomethylated genes

**Calcium signaling pathway**	**MAPK signaling pathway**
Cytoskeleton in muscle cells	**Wnt signaling pathway**
**Wnt signaling pathway**	**Rap1signaling pathway**
**Hippo signaling pathway**	**Proteoglycans in cancer**
Growth hormone synthesis	**Breast cancer**
**Thyroid hormone signaling pathway**	Phospholipase D signaling pathway
GRH signaling pathway	Signaling pathways regulating pluripotency of stem cells
Long-term potentiation	Dilated cardiomyopathy
**Tyrosine metabolism**	Circadian entrainment
beta-Alanine metabolism	GRH signaling pathway

The table lists enriched pathways, and the pathways related to cancer (as identified in the KEGG analysis) highlighted in bold.

**Table 6.** GO pathway enrichment analysis of differentially hypermethylated and hypomethylated genes in the C57BL/6 strain.

C57BL/6 GO analysis of hypermethylated genes	C57BL/6 GO analysis of hypomethylated genes

Regulation of membrane potential	**Negative regulation of protein modification process**
**Cell junction assembly**	**Transmembrane receptor protein serine/threonine kinase signaling pathway**
**Mitotic cell cycle phase transition**	Negative regulation of phosphate metabolic process
Muscle system process	Negative regulation of phosphorylation
**Positive regulation of cell cycle**	Negative regulation of protein phosphorylation
**Cognition**	Dendrite development
Learning or memory	**Embryonic organ morphogenesis**
Cell cycle G1/S phase transition	Dephosphorylation
**G1/S transition of mitotic cell cycle**	**Regulation of cell**
**Protein localization to cell junction**	**Regulation of cell morphogenesis**

The table lists enriched biological processes, and pathways related to cancer are highlighted in bold.

**Table 7.** GO pathway enrichment analysis of differentially hypermethylated and hypomethylated genes in the FVBN strain.

FVBN GO analysis of hypermethylated genes	FVBN GO analysis of hypomethylated genes

**Gland development**	Lymphocyte differentiation
Regulation of developmental growth	**Cell junction assembly**
**Ameboidal-type cell migration**	Epithelial tube morphogenesis
Regulation of neurogenesis	Dendrite development
Muscle cell differentiation	**Small GTPase mediated signal transduction**
Dendrite development	Regulation of developmental growth
Regulation of GTPase activity	**Cell-substrate adhesion**
**Positive regulation of growth**	Regulation of GTPase activity
Regulation of synapse structure or activity	Embryonic organ morphogenesis
Regulation of synapse organization	Locomotory behavior

The table lists enriched biological processes, and pathways related to cancer are highlighted in bold.

## Data Availability

Data are contained within the article.

## References

[R1] Aherrera A 2023. Metal concentrations in e-cigarette aerosol samples: a comparison by device type and flavor. Environ Health Perspect. 131(12):127004. 10.1289/EHP11921 PMC10695266 38048100

[R2] Almagro J, Messal HA, Elosegui-Artola A, van Rheenen J, Behrens A. 2022. Tissue architecture in tumor initiation and progression. Trends Cancer. 8(6):494–505. 10.1016/j.trecan.2022.02.007 35300951

[R3] American Lung Association. 2024. E-Cigarettes & Vaping [accessed 2024 Oct 9]. https://www.lung.org/quit-smoking/e-cigarettes-vaping

[R4] Ashburner M 2000. Gene Ontology: tool for the unification of biology. Nat Genet. 25(1):25–29. 10.1038/75556 PMC3037419 10802651

[R5] Babar Q 2022. Novel epigenetic therapeutic strategies and targets in cancer. Biochim Biophys Acta Mol Basis Dis. 1868(12):166552. 10.1016/j.bbadis.2022.166552 36126898

[R6] Balfour DJK 2021. Balancing consideration of the risks and benefits of E-cigarettes. Am J Public Health. 111(9):1661–1672. 10.2105/AJPH.2021.306416 PMC8589069 34410826

[R7] Bibikova M 2011. High density DNA methylation array with single CpG site resolution. Genomics. 98(4): 288–295. 10.1016/j.ygeno.2011.07.007 21839163

[R8] Bootman MD, Bultynck G. 2020. Fundamentals of cellular calcium signaling: a primer. Cold Spring Harb Perspect Biol. 12(1):a038802. 10.1101/cshperspect.a038802 PMC6942118 31427372

[R9] Bustamante G 2018. Presence of the carcinogen N′-nitrosonornicotine in saliva of E-cigarette users. Chem Res Toxicol. 31(8):731–738. 10.1021/acs.chemrestox.8b00089 PMC8556657 30019582

[R10] Caliri AW, Caceres A, Tommasi S, Besaratinia A. 2020. Hypomethylation of LINE-1 repeat elements and global loss of DNA hydroxymethylation in vapers and smokers. Epigenetics. 15(8):816–829. 10.1080/15592294.2020.1724401 PMC7518689 31996072

[R11] Cardenas A 2022. Epigenome-wide association study and epigenetic age acceleration associated with cigarette smoking among Costa Rican adults. Sci Rep. 12(1):4277. 10.1038/s41598-022-08160-w PMC8917214 35277542

[R12] CDC. 2024a. E-cigarette use among youth [accessed 2024 Oct 9]. https://www.cdc.gov/tobacco/e-cigarettes/youth.html

[R13] CDC. 2024b. E-cigarette use among adults [accessed 2025 Feb 20]. https://www.cdc.gov/tobacco/e-cigarettes/adults.html

[R14] Choi JD, Lee J-S. 2013. Interplay between epigenetics and genetics in cancer. Genomics Inform. 11(4):164–173. 10.5808/GI.2013.11.4.164 PMC3897842 24465226

[R15] Cruz-Tapias P, Castiblanco J, Anaya J-M. 2013. Major histocompatibility complex: antigen processing and presentation. In: Anaya J-M, Shoenfeld Y, Rojas-Villarraga A, Levy RA, Cervera R, editors. Autoimmunity: from bench to bedside. El Rosario University Press. 29087650

[R16] Cui H 2015. DNA methyltransferase 3A promotes cell proliferation by silencing CDK inhibitor p18INK4C in gastric carcinogenesis. Sci Rep. 5(1):13781. 10.1038/srep13781 PMC4563369 26350239

[R17] Das D, Karthik N, Taneja R. 2021. Crosstalk between inflammatory signaling and methylation in cancer. Front Cell Dev Biol. 9:756458. 10.3389/fcell.2021.756458 PMC8652226 34901003

[R18] de la Calle-Fabregat C 2024. NF-jB and TET2 promote macrophage reprogramming in hypoxia that overrides the immunosuppressive effects of the tumor microenvironment. Sci Adv. 10(38):eadq5226. 10.1126/sciadv.adq5226 PMC11409945 39292770

[R19] Díaz-Piña DA, Rivera-Ramırez N, García-López G, Díaz NF, Molina-Hernández A. 2024. Calcium and neural stem cell proliferation. Int J Mol Sci. 25(7):4073. 10.3390/ijms25074073 PMC11012558 38612887

[R20] Ding W 2025. Roles of the CDCA gene family in breast carcinoma. Sci Prog. 108(1):368504241312305. 10.1177/00368504241312305 PMC11736775 39814554

[R21] Fang F, Andersen AM, Philibert R, Hancock DB. 2023. Epigenetic biomarkers for smoking cessation. Addict Neurosci. 6:100079. 10.1016/j.addicn.2023.100079 PMC10136056 37123087

[R22] Fu M 2022. The hippo signalling pathway and its implications in human health and diseases. Signal Transduct Target Ther. 7(1):376. 10.1038/s41392-022-01191-9 PMC9643504 36347846

[R23] Garcia-Prieto CA 2022. Validation of a DNA methylation microarray for 285,000 CpG sites in the mouse genome. Epigenetics. 17(12):1677–1685. 10.1080/15592294.2022.2053816 PMC9621044 35297293

[R24] Gentleman RC 2004. Bioconductor: open software development for computational biology and bioinformatics. Genome Biol. 5(10):r80. 10.1186/gb-2004-5-10-r80 PMC545600 15461798

[R25] González-Castrillón LM, Wurmser M, Öhlund D, Wilson SI. 2023. Dysregulation of core neurodevelopmental pathways—a common feature of cancers with perineural invasion. Front Genet. 14:1181775. 10.3389/fgene.2023.1181775 PMC10501147 37719704

[R26] Gordon T 2022. E-cigarette toxicology. Annu Rev Pharmacol Toxicol. 62(1):301–322. 10.1146/annurev-pharmtox-042921-084202 PMC9386787 34555289

[R27] Gordon T, Bosland M. 2009. Strain-dependent differences in susceptibility to lung cancer in inbred mice exposed to mainstream cigarette smoke. Cancer Lett. 275(2):213–220. 10.1016/j.canlet.2008.10.012 PMC2708971 19118942

[R28] Han B 2020. DNA methylation biomarkers for nasopharyngeal carcinoma. PLOS One. 15(4):e0230524. 10.1371/journal.pone.0230524 PMC7144954 32271791

[R29] Hao J, Liu T, Xiu Y, Yuan H, Xu D. 2023. High DNA methylation age deceleration defines an aggressive phenotype with immunoexclusion environments in endometrial carcinoma. Front Immunol. 14:1208223. 10.3389/fimmu.2023.1208223 PMC10303802 37388735

[R30] Harlid S, Xu Z, Panduri V, Sandler DP, Taylor JA. 2014. CpG sites associated with cigarette smoking: analysis of epigenome-wide data from the sister study. Environ Health Perspect. 122(7):673–678. 10.1289/ehp.1307480 PMC4080519 24704585

[R31] He B 2023. ROS induced the Rab26 promoter hypermethylation to promote cigarette smoking-induced airway epithelial inflammation of COPD through activation of MAPK signaling. Free Radic Biol Med. 195:359–370. 10.1016/j.freeradbiomed.2023.01.001 36610560

[R32] Herzog C 2024. Cigarette smoking and E-cigarette use induce shared DNA methylation changes linked to carcinogenesis. Cancer Res. 84(11):1898–1914. 10.1158/0008-5472.CAN-23-2957 PMC11148547 38503267

[R33] Hikisz P, Jacenik D. 2023. The tobacco smoke component, acrolein, as a major culprit in lung diseases and respiratory cancers: molecular mechanisms of acrolein cytotoxic activity. Cells. 12(6):879. 10.3390/cells12060879 PMC10047238 36980220

[R34] Horvath S, Raj K. 2018. DNA methylation-based biomarkers and the epigenetic clock theory of ageing. Nat Rev Genet. 19(6):371–384. 10.1038/s41576-018-0004-3 29643443

[R35] Horvath S 2013. DNA methylation age of human tissues and cell types. Genome Biol. 14(10):R115. 10.1186/gb-2013-14-10-r115 PMC4015143 24138928

[R36] Hubert KA, Wellik DM. 2023. Hox genes in development and beyond. Development. 150(1):dev192476. 10.1242/dev.192476 PMC10216783 36645372

[R37] Huynh D 2020. Electronic cigarettes promotes the lung colonization of human breast cancer in NOD-SCID-gamma mice. Int J Clin Exp Pathol. 13(8):2075–2081. PMC7476960 32922603

[R38] Jin H 2015. NNK-induced DNA methyltransferase 1 in lung tumorigenesis in A/J mice and inhibitory effects of (−)-epigallocatechin-3-gallate. Nutr Cancer. 67(1):167–176. 10.1080/01635581.2015.976314. PMC4363938 25437343

[R39] Joehanes R 2016. Epigenetic signatures of cigarette smoking. Circ Cardiovasc Genet. 9(5):436–447. 10.1161/CIRCGENETICS.116.001506 PMC5267325 27651444

[R40] Joubert BR 2012. 450K epigenome-wide scan identifies differential DNA methylation in newborns related to maternal smoking during pregnancy. Environ Health Perspect. 120(10):1425–1431. 10.1289/ehp.1205412. PMC3491949 22851337

[R41] Kankanamage RNT 2020. Metabolites of tobacco- and E-cigarette-related nitrosamines can drive Cu^2+^-mediated DNA oxidation. Chem Res Toxicol. 33(8):2072–2086. 10.1021/acs.chemrestox.0c00027 PMC7510339 32672941

[R42] Keerthana CK 2023. The role of AMPK in cancer metabolism and its impact on the immunomodulation of the tumor microenvironment. Front Immunol. 14: 1114582. 10.3389/fimmu.2023.1114582 PMC9975160 36875093

[R43] Kosumi K 2020. Tumor long-interspersed nucleotide element-1 methylation level and immune response to esophageal cancer. Ann Surg. 272(6):1025–1034. 10.1097/SLA.0000000000003264 30946079

[R44] Kota NK, Vigorito M, Krishnan V, Chang SL. 2022. Using IPA tools to characterize molecular pathways underlying the involvement of IRF7 in antiviral response to HIV. NeuroImmune Pharm Ther. 1(1):23–35. 10.1515/nipt-2022-0009 PMC9923504 36827648

[R45] Kresovich JK 2019. Methylation-based biological age and breast cancer risk. J Natl Cancer Inst. 111(10):1051–1058. 10.1093/jnci/djz020 PMC6792078 30794318

[R46] Lampos S 2019. Real-time assessment of E-cigarettes and conventional cigarettes emissions: aerosol size distributions, mass and number concentrations. Toxics. 7(3): 45. 10.3390/toxics7030045 PMC6789668 31480224

[R47] Lee KWK, Pausova Z. 2013. Cigarette smoking and DNA methylation. Front Genet. 4:132. 10.3389/fgene.2013.00132 PMC3713237 23882278

[R48] Levine ME 2018. An epigenetic biomarker of aging for lifespan and healthspan. Aging. 10(4):573–591. 10.18632/aging.101414 PMC5940111 29676998

[R49] Lewis SM, Williams A, Eisenbarth SC. 2019. Structure–function of the immune system in the spleen. Sci Immunol. 4(33):eaau6085. 10.1126/sciimmunol.aau6085 PMC6495537 30824527

[R50] Li J 2022. Electronic cigarettes versus nicotine-replacement therapy for smoking cessation: a systematic review and meta-analysis of randomized controlled trials. Tob Induc Dis. 20:90. 10.18332/tid/154075 PMC9582581 36339933

[R51] Li L, Lee ES, Nguyen C, Zhu Y. 2020. Effects of propylene glycol, vegetable glycerin, and nicotine on emissions and dynamics of electronic cigarette aerosols. Aerosol Sci Technol. 54(11):1270–1281. 10.1080/02786826.2020.1771270 PMC7590927 33116348

[R52] Lin R-K 2010. The tobacco-specific carcinogen NNK induces DNA methyltransferase 1 accumulation and tumor suppressor gene hypermethylation in mice and lung cancer patients. J Clin Invest. 120(2):521–532. 10.1172/JCI40706. PMC2810088 20093774

[R53] Ma Y, Li MD. 2017. Establishment of a strong link between smoking and cancer pathogenesis through DNA methylation analysis. Sci Rep. 7:1811. 10.1038/s41598-017-01856-4. PMC5431893 28500316

[R54] Li Q 2024. Signaling pathways involved in colorectal cancer: pathogenesis and targeted therapy. Signal Transduct Target Ther. 9(1):266. 10.1038/s41392-024-01953-7 PMC11456611 39370455

[R55] Li S 2024. Integrated analysis of the DNA methylome and RNA transcriptome during the development of skeletal muscle in Duroc pigs. BMC Genomics. 25(1):504. 10.1186/s12864-024-10404-0 PMC11110227 38778260

[R56] Maksimovic J, Oshlack A, Phipson B. 2021. Gene set enrichment analysis for genome-wide DNA methylation data. Genome Biol. 22(1):173. 10.1186/s13059-021-02388-x PMC8186068 34103055

[R57] Merecz-Sadowska A 2020. A summary of in vitro and in vivo studies evaluating the impact of E-cigarette exposure on living organisms and the environment. Int J Mol Sci. 21(2):652. 10.3390/ijms21020652 PMC7013895 31963832

[R58] Molony RD, Wu C-H, Lee Y-F. 2023. E-liquid exposure induces bladder cancer cells to release extracellular vesicles that promote non-malignant urothelial cell transformation. Sci Rep. 13(1):142. 10.1038/s41598-022-27165-PMC9813241 36599909

[R59] Mori KM 2022. Lung mitochondrial DNA copy number, inflammatory biomarkers, gene transcription and gene methylation in vapers and smokers. EBioMedicine. 85:104301. 10.1016/j.ebiom.2022.104301 PMC9561685 36215783

[R60] Nadziejko C, Fang K, Bravo A, Gordon T. 2007. Susceptibility to pulmonary hypertension in inbred strains of mice exposed to cigarette smoke. J Appl Physiol. 102(5):1780–1785. 10.1152/japplphysiol.01076.2005 17272409

[R61] Nakata Y 2024. PTIP epigenetically regulates DNA damage-induced cell cycle arrest by upregulating PRDM1. Sci Rep. 14(1):17987. 10.1038/s41598-024-68295-w PMC11297997 39097652

[R62] Ogunwale MA 2017. Aldehyde detection in electronic cigarette aerosols. ACS Omega. 2(3):1207–1214. 10.1021/acsomega.6b00489 PMC5377270 28393137

[R63] Patil N 2021. Changes in methylation across structural and microRNA genes relevant for progression and metastasis in colorectal cancer. Cancers. 13(23):5951. 10.3390/cancers13235951 PMC8656781 34885060

[R64] Payne JD, Orellana-Barrios M, Medrano-Juarez R, Buscemi D, Nugent K. 2016. Electronic cigarettes in the media. Proc Bayl Univ Med Cent. 29:280–283. 10.1080/08998280.2016.11929436 PMC4900769 27365871

[R65] Pidsley R 2016. Critical evaluation of the Illumina MethylationEPIC BeadChip microarray for whole-genome DNA methylation profiling. Genome Biol. 17(1): 208. 10.1186/s13059-016-1066-1 PMC5055731 27717381

[R66] Qi H, Song S, Wang P. 2022. ImmuMethy, a database of DNA methylation plasticity at a single cytosine resolution in human blood and immune cells. Database. 2022:baac020. 10.1093/database/baac020 PMC9216548 35363305

[R67] Richmond RC 2021. Investigating the DNA methylation profile of E-cigarette use. Clin Epigenet. 13(1):183. 10.1186/s13148-021-01174-7 PMC8479883 34583751

[R68] Sahoo K, Sundararajan V. 2024. Methods in DNA methylation array dataset analysis: a review. Comput Struct Biotechnol J. 23:2304–2325. 10.1016/j.csbj.2024.05.015 PMC11153885 38845821

[R69] Sanchez-Vega F 2018. Oncogenic signaling pathways in The Cancer Genome Atlas. Cell. 173(2):321–337.e10. 10.1016/j.cell.2018.03.035 PMC6070353 29625050

[R70] Saunders AM, Seldin MF. 1990. A molecular genetic linkage map of mouse chromosome 7. Genomics. 8(3):525–535. 10.1016/0888-7543(90)90040-2 1981054

[R71] Sethi JK, Vidal-Puig A. 2010. Wnt signalling and the control of cellular metabolism. Biochem J. 427(1):1–17. 10.1042/BJ20091866 PMC4301310 20226003

[R72] Sever R, Brugge JS. 2015. Signal transduction in cancer. Cold Spring Harb. Perspect. Med. 5:a006098. 10.1101/cshperspect.a006098 PMC4382731 25833940

[R73] Sharma A, McCausland K, Jancey J. 2021. Adolescent’s health perceptions of E-cigarettes: a systematic review. Am J Prev Med. 60(5):716–725. 10.1016/j.amepre.2020.12.013 33775514

[R74] Shehata SA 2023. Vaping, environmental toxicants exposure, and lung cancer risk. Cancers. 15(18):4525. 10.3390/cancers15184525 PMC10526315 37760496

[R75] Song M-A 2023. Accelerated epigenetic age, inflammation, and gene expression in lung: comparisons of smokers and vapers with non-smokers. Clin Epigenet. 15(1):160. 10.1186/s13148-023-01577-8 PMC10568901 37821974

[R76] Su J 2024. Cell–cell communication: new insights and clinical implications. Signal Transduct. Target. Ther. 9(1–52):196. 10.1038/s41392-024-01888-z PMC11382761 39107318

[R77] Suthar SK, Lee S-Y. 2022. Ingenuity pathway analysis of α-synuclein predicts potential signaling pathways, network molecules, biological functions, and its role in neurological diseases. Front Mol Neurosci. 15:1029682. 10.3389/fnmol.2022.1029682 PMC9744789 36523604

[R78] Taylor KM, 2018. The tobacco carcinogen NNK drives accumulation of DNMT1 at theGR promoter thereby reducing GR expression in untransformed lung fibroblasts. Sci Rep. 8:4903. 10.1038/s41598-018-23309-2. PMC5861118 29559689

[R79] Tommasi S 2019. Deregulation of biologically significant genes and associated molecular pathways in the oral epithelium of electronic cigarette users. Int J Mol Sci. 20(3):738. 10.3390/ijms20030738 PMC6386888 30744164

[R80] Tripathi BK 2024. The pro-oncogenic noncanonical activity of a RASGTP: RanGAP1 complex facilitates nuclear protein export. Nat Cancer. 5(12):1902–1918. 10.1038/s43018-024-00847-5 PMC11663792 39528835

[R81] Vidaki A 2023. Targeted DNA methylation analysis and prediction of smoking habits in blood based on massively parallel sequencing. Forensic Sci Int Genet. 65: 102878. 10.1016/j.fsigen.2023.102878 37116245

[R82] Wang J 2012. The tobacco-specific carcinogen NNK induces DNA methyltransferase 1 accumulation in laryngeal carcinoma. Oral Oncol. 48(6):541–546. 10.1016/j.oraloncology.2012.01.007 22317856

[R83] Wang K 2022. Epigenetic regulation of aging: implications for interventions of aging and diseases. Signal Transduct Target Ther. 7(1):374. 10.1038/s41392-022-01211-8 PMC9637765 36336680

[R84] Wang L, Wang Y, Chen J, Liu P, Li M. 2022. A review of toxicity mechanism studies of electronic cigarettes on respiratory system. Int J Mol Sci. 23(9):5030. 10.3390/ijms23095030 PMC9102406 35563421

[R85] Wang L-J, Xu R, Wu Y. 2024. Migrasome regulator TSPAN4 shapes the suppressive tumor immune microenvironment in pan-cancer. Front Immunol. 15:1419420. 10.3389/fimmu.2024.1419420 PMC11668678 39723210

[R86] Wu H 2024. Emerging mechanisms and promising approaches in pancreatic cancer metabolism. Cell Death Dis. 15(8):553. 10.1038/s41419-024-06930-0 PMC11294586 39090116

[R87] Xiao Y, Word B, Lyn-Cook L, Lyn-Cook B, Hammons G. 2015. Cigarette smoke condensate and individual constituents modulate DNA methyltransferase expression in human liver cells. SAGE Open Med. 3:2050312115578317. 10.1177/2050312115578317 PMC4679232 26770776

[R88] Xie Z, Rahman I, Goniewicz ML, Li D. 2021. Perspectives on epigenetics alterations associated with smoking and vaping. Function. 2(3):zqab022. 10.1093/function/zqab022 PMC8788872 35330676

[R89] Zamora Goicoechea J 2024. A global health survey of people who vape but never smoked: protocol for the VERITAS (Vaping Effects: real-World International Surveillance) Study. JMIR Res Protoc. 13:e54236. 10.2196/54236 PMC11009848 38546715

[R90] Zhang B 2023. WIF1 promoter hypermethylation induce endometrial carcinogenesis through the Wnt/β-catenin signaling pathway. Am J Reprod Immunol. 90(2):e13743. 10.1111/aji.13743 37491917

[R91] Zhang M, Hu T, Ma T, Huang W, Wang Y. 2024. Epigenetics and environmental health. Front Med. 18(4): 571–596. 10.1007/s11684-023-1038-2 38806988

[R92] Zhao H 2022. Wnt signaling in colorectal cancer: pathogenic role and therapeutic target. Mol Cancer. 21(1):144. 10.1186/s12943-022-01616-7 PMC9281132 35836256

[R93] Zhou W 2022. DNA methylation dynamics and dysregulation delineated by high-throughput profiling in the mouse. Cell Genom. 2(7):100144. 10.1016/j.xgen.2022.100144 PMC9306256 35873672

[R94] Zhou W, Triche TJ, Laird PW, Shen H. 2018. SeSAMe: reducing artifactual detection of DNA methylation by Infinium BeadChips in genomic deletions. Nucleic Acids Res. 46(20):e123. 10.1093/nar/gky691 PMC6237738 30085201

[R95] Zong D, Liu X, Li J, Ouyang R, Chen P. 2019. The role of cigarette smoke-induced epigenetic alterations in inflammation. Epigenet Chrom. 12(1):65. 10.1186/s13072-019-0311-8 PMC6844059 31711545

