# Supplementary material for: Exposure to electronic cigarette aerosols triggers alterations in genomic DNA methylation that impacts cancer pathways in mice
Source: Inhal Toxicol. Author manuscript; Available in PMC 2026 Oct 1. (PMC13629434; doi:10.1080/08958378.2026.2649516)
Supplement: Supplementary file 1 [file NIHMS2171332-supplement-Supp_1.docx]

**Supplemental Fig 1.** Principal Component Analysis (PCA) was performed to visualize the variance in DNA methylation patterns in: (a) C57BL/6; and (b) FVBN mice. Each individual plot demonstrates the separation or clustering of the two mouse strains based on their methylation profiles.
